# The long noncoding RNA *mimi* scaffolds neuronal granules to maintain nervous system maturity

**DOI:** 10.1126/sciadv.abo5578

**Published:** 2022-09-28

**Authors:** Dominika Grzejda, Jana Mach, Johanna Aurelia Schweizer, Barbara Hummel, Andrew Mischa Rezansoff, Florian Eggenhofer, Amol Panhale, Maria-Eleni Lalioti, Nina Cabezas Wallscheid, Rolf Backofen, Johannes Felsenberg, Valérie Hilgers

**Affiliations:** ^1^ Max-Planck-Institute of Immunobiology and Epigenetics, Freiburg 79108, Germany.; ^2^ Faculty of Biology, Albert Ludwig University of Freiburg, Freiburg 79104, Germany.; ^3^ International Max Planck Research School for Molecular and Cellular Biology (IMPRS- MCB), Freiburg 79108, Germany.; ^4^ Friedrich Miescher Institute for Biomedical Research (FMI), Basel 4058, Switzerland.; ^5^ University of Basel, Basel 4001, Switzerland.; ^6^ Department of Computer Science, Albert Ludwig University of Freiburg, Freiburg 79110, Germany.; ^7^ BIOSS and CIBSS Centres for Biological Signalling Studies, University of Freiburg, Freiburg 79104, Germany.; ^8^ CIBSS Centre for Integrative Biological Signalling Studies, University of Freiburg, Freiburg 79104, Germany.

## Abstract

RNA binding proteins and messenger RNAs (mRNAs) assemble into ribonucleoprotein granules that regulate mRNA trafficking, local translation, and turnover. The dysregulation of RNA-protein condensation disturbs synaptic plasticity and neuron survival and has been widely associated with human neurological disease. Neuronal granules are thought to condense around particular proteins that dictate the identity and composition of each granule type. Here, we show in *Drosophila* that a previously uncharacterized long noncoding RNA, *mimi*, is required to scaffold large neuronal granules in the adult nervous system. Neuronal ELAV-like proteins directly bind *mimi* and mediate granule assembly, while Staufen maintains condensate integrity. *mimi* granules contain mRNAs and proteins involved in synaptic processes; granule loss in *mimi* mutant flies impairs nervous system maturity and neuropeptide-mediated signaling and causes phenotypes of neurodegeneration. Our work reports an architectural RNA for a neuronal granule and provides a handle to interrogate functions of a condensate independently of those of its constituent proteins.

## Teaser

The condensation of neuronal RNA binding proteins around the arcRNA *mimi* is critical for adult nervous system function.

## INTRODUCTION

Cells partition their content as a strategy to coordinate the function of biomolecules in space and time. Ribonucleoprotein (RNP) granules are membrane-less cellular compartments composed of RNA and RNA binding proteins (RBPs) that interact with each other through multivalent interactions and dynamically exchange with the surrounding cellular milieu (*1*). In the nucleus, granules are often scaffolded by long noncoding RNAs (lncRNAs), termed architectural RNAs (arcRNAs). In contrast, cytoplasmic granules are typically characterized by the presence of specific proteins. Cytoplasmic compartmentalization via biomolecular condensation is evolutionarily ancient and supports the maintenance of cellular function in a broad range of tissues; however, diseases linked to RNP granule homeostasis are predominantly associated with the nervous system. Hypo-assembly of RNP granule components in RBP loss-of-function models has been linked to neurodevelopmental, neurodegenerative, and neuropsychiatric disorders, while granule hyper-assembly and maturation into aggregates notoriously cause neurodegenerative diseases (*2*, *3*).

The members of the highly conserved Staufen family of RBPs (*4*) compartmentalize the neuronal cytoplasm by forming two distinct types of granules: large, mostly immobile condensates associated with the rough endoplasmatic reticulum membrane and small granules that actively transport mRNAs from the soma to dendrites (*5*–*9*). *Drosophila* and mouse *staufen* mutants display severe neurological defects, including abnormal dendritic arborization, reduced locomotor activity, and memory deficits (*10*–*12*). The role of each type of Staufen condensate and the respective contribution to these phenotypes are not understood. Another well-known family of granule-associated RBPs comprises the highly conserved neuronal ELAV (embryonic lethal abnormal vision)–like proteins (nELAV proteins), which serve as early markers of neuronal identity across model organisms (*13*). Mammalian and *Drosophila* cytoplasmic nELAVs play important roles in neuronal development, synaptogenesis, and synaptic plasticity/excitability (*14*–*16*). nELAV-deficient animals display a wide range of neurological phenotypes including epileptic seizures, impaired behavior, and age-dependent progressive motor deficits (*17*–*19*). Cytoplasmic nELAV proteins are found in different types of RNP granules, including neuronal granules (*20*), stress granules (*21*), and pathogenic insoluble inclusions (*22*). How the cellular functions of nELAV proteins are tied to their condensation in the neuronal cytoplasm has not been established.

Contrasting with the prevailing model that cytoplasmic granules are scaffolded by proteins, in this study, we describe the first arcRNA for neuronal granules: the previously uncharacterized lncRNA *mimi*. *mimi* is undetectable outside of condensates, suggesting that its main cellular function is to support RNP granules. In addition, we find that Staufen, nELAV proteins, and *mimi* are all essential for those granules, demonstrating specific and nonredundant interactions at the core of multicomponent condensates. Our findings show how the molecular functions of nELAV proteins and Staufen converge in *mimi* condensates in the adult brain to regulate neuronal signaling and behavior.

## RESULTS

### FNE and RBP9 are required for specific neuronal granules

**Fig. 1.**
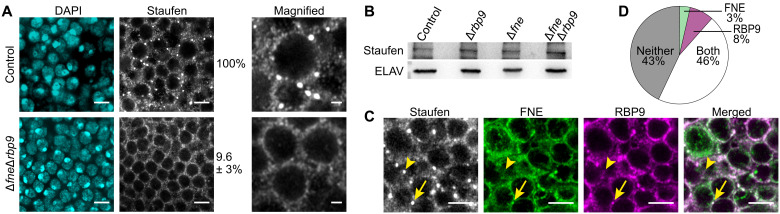
FNE and RBP9 are required for large Stau granules in neurons. (**A**) Confocal imaging of neurons in the midbrain of control (*w^1118^*) and Δ*fne*Δ*rbp9* adult flies. The number of large Stau granules is indicated as a percentage of granules per cell found in control brains. Cells scored: *n* = 550 (control) and *n* = 645 (Δ*fne*Δ*rbp9*). DAPI, 4′,6-diamidino-2-phenylindole. (**B**) Western blot comparing Stau protein expression in adult fly heads in the indicated genotypes. ELAV serves as a loading control. (**C** and **D**) Confocal imaging (C) and quantification (D) of the association of large Stau granules with tag-FNE and tag-RBP9. Granules typically contained Stau only (arrowhead) or all three proteins (arrow). Granules scored: *n* = 376. Scale bars, 4 μm (magnified images, 1 μm).

We investigated whether FNE and RBP9, the two cytoplasmic *Drosophila* nELAV proteins, are involved in neuronal granule formation or maintenance. We assessed the morphology and abundance of different types of RNP granules in the brain of adult flies lacking FNE (Δ*fne*), RBP9 (Δ*rbp9*), or both (Δ*fne*Δ*rbp9*). Granules marked with *Drosophila* Imp (*23*), Trailer Hitch (Tral), or Fragile X mental retardation protein (FRMP) (*7*) were not detectably affected in neurons of Δ*fne*Δ*rbp9* flies (fig. S1A). However, large Staufen (Stau) granules were drastically and specifically depleted (Fig. 1A). Small Stau granules were preserved, and Stau protein levels were not affected (Fig. 1, A and B), indicating that Stau underwent redistribution from granules to the neuronal cytoplasm. Moreover, large Stau granules were not depleted in individual Δ*fne* or Δ*rbp9* mutants (fig. S1B). Together, our results show that FNE and RBP9 act redundantly in forming or preserving large Stau granules.

Next, to detect FNE and RBP9 by immunofluorescence (IF), we N-terminally tagged the endogenous *fne* (*24*) and *rbp9* loci with FLAG-V5 and FLAG-MYC, respectively (fig. S1, C and D). Imaging of MYC, V5, and Stau in adult brains revealed that roughly half of large Stau granules include tag-FNE and tag-RBP9. Given that tag-RBP9 is expressed at levels similar to tag-FNE (fig. S1E), it is possible that Stau granules contain comparable levels of RBP9 and FNE. Notably, Stau granules contained either both or neither of the two proteins (Fig. 1, C and D, and fig. S1F). Together, our data show that FNE and RBP9 colocalize in Stau granules and indicate the existence of a possible intermediate molecule through which the three RBPs interact.

### FNE and RBP9 regulate a lncRNA specific to the adult brain: *mimi*

**Fig. 2.**
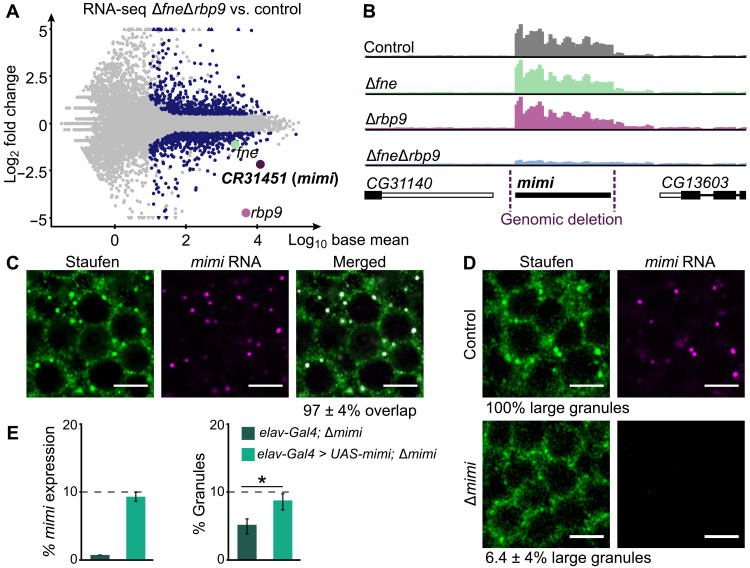
*mimi* is an arcRNA for large Stau granules. (**A**) Differential RNA expression in Δ*fne*Δ*rbp9* mutants compared to control flies (*w^1118^*) represented as a function of mean expression levels. Dark blue represents: |log_2_ fold change| > 0.5 with *P* < 0.01 and base mean > 10. (**B**) RNA-seq tracks for *mimi* and flanking gene regions in the indicated genotypes. The genomic deletion in Δ*mimi* flies is indicated. Range: 0 to 1864 reads per kilobase million (RPKM). (**C** and **D**) Confocal imaging of Stau IF combined with *mimi* RNA in situ hybridization. Quantifications represent large Stau granules that contain *mimi* in wild-type neurons (*w^1118^*) (granules scored: *n* = 386) (C) and large Stau granules found in Δ*mimi* brains compared to control (*w^1118^*) [cells scored: *n* = 429 (control) and *n* = 445 (Δ*mimi*)] (D). Scale bars, 4 μm. (**E**) Quantification of *mimi* expression and granule number in Δ*mimi* mutants (*elav-Gal4;* Δ*mimi*) and Δ*mimi* mutants in which *mimi* was expressed from a transgene (*elav-Gal4 > UAS-mimi;* Δ*mimi*). Reverse transcription quantitative polymerase chain reaction (RT-qPCR) signal was normalized to *RpL32*. Expression and granule number in wild-type flies are set to 100%. Error bars represent ±SD of three biological replicates. Cells scored for granule quantification: *n* = 731 (control) and *n* = 623 (rescue). *P = 0.032 (two-sided *t* test).

Because FNE and RBP9 are required for Stau granule formation but do not appear to be systematically associated with them, we considered the possibility that FNE and RBP9 regulate a granule constituent RNA. To test this possibility, we performed protein immunoprecipitation (IP) from head tissue of flies coexpressing FLAG-V5-FNE and FLAG-MYC-RBP9, using antibodies directed against V5 and MYC, in the presence and absence of ribonuclease (RNase). We found that tag-FNE and tag-RBP9 interact in a predominantly RNA-dependent manner (fig. S1G). Therefore, we set out to identify an RNA coregulated by FNE and RBP9 in granules. Total RNA sequencing (RNA-seq) on adult fly heads revealed the lncRNA *CR31451* as the top down-regulated transcript in Δ*fne*Δ*rbp9* (Fig. 2A). We set out to further characterize *CR31451* and named it “*mimi*” (French for “cute”). *mimi* is a polyadenylated, ≈1-kb lncRNA (fig. S2, A and B). Notably, *mimi-RC* (thereafter referred to simply as *mimi*) is exclusively restricted to the adult nervous system and constitutes one of the most highly expressed polyadenylated RNAs in the *Drosophila* head (Fig. 2A and fig. S2, C and D). In Δ*fne*Δ*rbp9* mutants, the selective depletion of *mimi* (Fig. 2B and fig. S2E) concurrently with the specific loss of large Stau condensates (Fig. 1A), raises the possibility that *mimi* constitutes an architectural component of those granules.

### *mimi* is an arcRNA for large Stau granules

To qualify as an arcRNA for an RNP granule, a lncRNA needs to fulfill two requirements: (i) enrichment in specific biomolecular condensates and (ii) disintegration of the condensates and dispersion of protein markers upon its removal. First, we determined *mimi* subcellular localization. In adult *Drosophila* brains, *mimi* was exclusively restricted to large Stau condensates and absent from small Stau granules and from the cytoplasm (Fig. 2C and fig. S2, F to H). Therefore, *mimi* is a specific, stable component of large Stau condensates. Second, we generated a fly specifically lacking *mimi* (Fig. 2B). Notably, adult Δ*mimi* brains were completely devoid of large Stau granules. Loss of *mimi* caused a redistribution of Stau into the neuronal cytoplasm but did not alter total Stau protein levels (Fig. 2D and fig. S2I). tag-FNE and tag-RBP9 were up-regulated but expressed in the expected cytoplasmic pattern (fig. S2, J and K). Next, we investigated whether reintroduction of *mimi* RNA into Δ*mimi* brains can restore *mimi* granule formation. Because *mimi* RNA is very highly expressed endogenously (Fig. 2A), our genetic rescue experiment used a *UAS-mimi* transgene under the control of a strong, pan-neuronal, *elav-GAL4* driver. Nonetheless, we were able to reach only ≈10% of wild-type *mimi* RNA levels in Δ*mimi* flies, which restored *mimi* granules to 9% of their wild-type levels (Fig. 2E). This partial rescue confirms that granule loss in Δ*mimi* flies is caused by the absence of *mimi* and suggests that the lncRNA constitutes a limiting factor for granule formation. In conclusion, *mimi* is the first described arcRNA for neuronal granules: “*mimi* granules,” large Stau-containing cytoplasmic condensates characteristic of neurons of the adult nervous system.

### *mimi* is directly bound by FNE, RBP9, and Stau in vivo

**Fig. 3.**
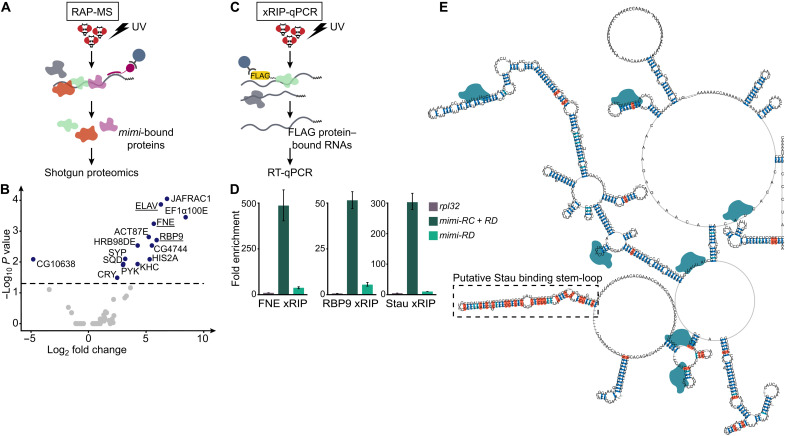
*mimi* is directly bound by FNE, RBP9, and Stau in vivo. (**A**) RAP-MS workflow. *mimi* RNA was hybridized to biotinylated DNA probes and captured on streptavidin beads. UV–cross-linked protein-RNA complexes were analyzed by shotgun proteomics. (**B**) Proteins recovered by *mimi* RAP-MS. In blue, significantly enriched or depleted proteins compared to the non–cross-linked control (*P* < 0.05, two-sided limma *t* test adjusted with the Benjamini-Yekutieli method). nELAV family proteins are underlined. (**C**) xRIP-qPCR workflow. tag-FNE, tag-RBP9, and tag-Stau proteins were captured on anti-FLAG beads. UV–cross-linked protein-RNA complexes were analyzed by RT-qPCR. (**D**) RT-qPCR quantification of RNA levels of the indicated transcripts in FLAG-xRIP samples from flies expressing tag-FNE, tag-RBP9, or tag-Stau. The RNA fold enrichment in the xRIP-tagged sample over input was normalized to enrichment in the untagged control (*w^1118^*) for each genotype. Error bars represent ±SD of at least three biological replicates for each genotype. (**E**) Consensus secondary structure predicted for *mimi* RNA based on multiple sequence-structure alignment. Paired nucleotides with increased covariance are in orange. Putative Stau, FNE, and RBP9 binding is indicated as a dotted line box and blue shapes, respectively.

We tested whether permanent (Stau) and dynamic (FNE and RBP9) *mimi* granule components directly bind *mimi*. First, we identified *mimi*-bound proteins by applying RNA antisense purification and mass spectrometry (RAP-MS) (*25*) to adult fly heads (Fig. 3A and fig. S3, A and B). We identified 14 proteins specifically enriched in the cross-linked sample, among which all three *Drosophila* nELAV proteins (ELAV, FNE, and RBP9) were top hits (Fig. 3B). While FNE and RBP9 interact with *mimi* in cytoplasmic granules, the exclusively nuclear protein ELAV does not colocalize with *mimi* granules (fig. S3C) and likely binds *mimi* before the RNA exits the nucleus; alternatively, the identified nuclear proteins may constitute contaminants of the sample. Stau could not be detected in any sample, including the input, likely due to the low sensitivity of shotgun proteomics. To assess whether Stau directly binds *mimi* and confirm results for FNE and RBP9, we performed RNA immunoprecipitation with ultraviolet (UV) cross-linking (xRIP) on the head tissue of flies expressing, from the endogenous locus, tag-Stau, tag-FNE, or tag-RBP9 (Fig. 3C and figs. S1, C and D, and S3, D and E). We observed high and specific enrichment of *mimi* in the protein-bound RNA fraction for all three RBPs (Fig. 3D). Together, our results demonstrate that Stau and *mimi* directly interact to constitute *mimi* granules. FNE and RBP9 bind *mimi* directly and regulate the formation and/or maintenance of *mimi* granules.

To better understand the RNA-protein interactions at the source of *mimi* granules, we combined *mimi* secondary structure prediction with a covariance analysis that describes how distinct nucleotides have coevolved to maintain structural elements (fig. S3F) and generated a structural model of *mimi* (Fig. 3E). Because nELAV protein family members can interchangeably act on the same RNA target sequences (*26*), and ELAV directly and specifically binds *mimi* (Fig. 3B and fig. S3G), we consider *mimi* regions identified in ELAV iCLIP (individual-nucleotide resolution UV crosslinking and immunoprecipitation) (*24*) to be putative FNE and/or RBP9 binding sites. Notably, ELAV binding predominantly occurs at predicted loop regions (Fig. 3E), consistent with ELAV’s known binding to single-stranded RNA (*27*). Stau reportedly interacts with RNAs through well-described secondary structures (*28*, *29*). In the *mimi* structural model, one region stood out by its increased covariance among homologs, thereby possibly representing a secondary structure of conserved functional relevance. Notably, this region is a stereotypical Stau binding stem-loop (Fig. 3E), which we propose constitutes the main interaction site for Stau.

### Granule assembly and maturation depend on *mimi* interaction with FNE, RBP9, and Stau

**Fig. 4.**
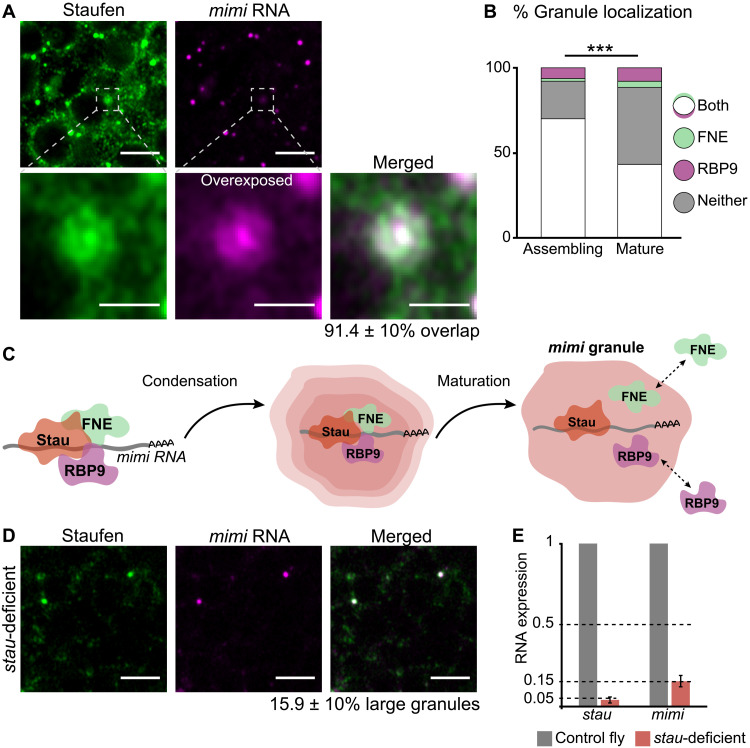
Granule assembly and maturation depend on *mimi* interaction with FNE, RBP9, and Stau. (**A**) Visualization of *mimi* and Stau accumulation, and quantification of signal overlap, in precursor structures of cytoplasmic condensates in neurons of wild-type flies. *mimi* signal is overexposed for visibility. Scale bars, 4 μm (magnified images, 1 μm). (**B**) Quantification of the association of diffuse (assembling) and condensed (mature) Stau foci with tag-FNE and tag-RBP9. Data for mature granules are reproduced from Fig. 1. ***P = 3.7 × 10^−4^ (two-sided Fisher’s exact test); assembling granules scored: *n* = 72 (A) and *n* = 63 (B). (**C**) Model of *mimi* granule assembly. Local interactions of *mimi* with FNE, RBP9, and Stau are essential to initiate granule assembly. Interactions with FNE and RBP9 decrease with granule maturation, whereas Stau and *mimi* remain constitutive granule components. (**D**) Confocal imaging of neurons in the midbrain of *stau*-deficient adult flies and quantification of granule number. Cells scored: *n* = 429 (*w^1118^* control, not shown) and *n* = 487 (*stau*-deficient). Scale bars, 4 μm. (**E**) RT-qPCR quantification of *stau* and *mimi* RNA levels in control (*w^1118^*) and *stau*-deficient adult heads. Signal was normalized to *RpL32*. Error bars represent ±SD of four biological replicates.

The formation of mature RNP granules is preceded by a local increase in concentration of proteins and associated RNAs (*30*). In fly brains, we were able to visualize structures that likely represent a precursor state of *mimi* granules: cytoplasmic foci in which Stau and *mimi* signal colocalize in a concentric pattern (Fig. 4A). Notably, tag-FNE and tag-RBP9 were found in these less condensed foci significantly more frequently than in mature granules (Fig. 4B and fig. S4A), suggesting that FNE and RBP9 are required for granule assembly rather than maintenance. To test whether the role of nELAV proteins in neuronal granule regulation is conserved in mammals, we cultured cells from the subventricular zone of adult mice and visualized nELAV/Hu proteins and Stau1 in differentiated neurons (fig. S4B). We found that roughly one-third of large Stau1 granules contained HuB, HuC, and HuD, respectively (fig. S4C), suggesting a potential role for mammalian nELAV proteins in Stau1 granule assembly. We propose the following model: Stau and nELAV proteins directly bind to *mimi* and instruct the assembly of granules. *mimi* and Stau constitute the core of mature granules, whereas nELAV proteins interact in a dynamic fashion. In *mimi* mutant flies, loss of the interactions between Stau, FNE, RBP9, and *mimi* prevents granule formation (Fig. 4C).

Consistent with Stau, FNE, and RBP9 expression in both developing and mature neurons and their broad localization pattern throughout the cytoplasm, all three proteins are required for multiple aspects of neuron physiology (*4*, *19*, *31*); so far, it has not been possible to characterize adult-specific roles or to uncouple granule-dependent from granule-independent functions of the three RBPs. In contrast, *mimi* has the potential to represent the unique link between FNE, RBP9, and Stau functions in the condensates of mature nervous system. To address how exclusive *mimi* is to those condensates, we investigated whether the lncRNA can exist outside of granules. We used a combination of two alleles to obtain a fly in which *stau* levels were strongly reduced (fig. S4D). This eliminated 85% of *mimi* granules (Fig. 4D) and decreased *mimi* RNA levels by 85% (Fig. 4E). Residual *mimi* RNAs were exclusively found in granules marked with residual Stau protein (Fig. 4D and fig. S4E). tag-FNE and tag-RBP9 proteins were up-regulated (fig. S4F), indicating that, although necessary (Fig. 2), the two RBPs are not sufficient for *mimi* expression. Although we cannot exclude a possible effect of FNE, RBP9, or Stau loss on *mimi* transcription or nuclear export, our data, together with the depletion of *mimi* in an independent model of granule loss (Δ*fne*Δ*rbp9* mutant; Fig. 1A), strongly suggest that *mimi* can only subsist when incorporated into granules, where it is possibly protected from cytosolic microRNAs or RBPs by the phase boundary. Because *mimi* is required for *mimi* granules, Δ*mimi* mutant flies represent a unique animal model of condensate loss and give us the opportunity to study the composition and physiological function of *mimi* granules in vivo, independently of its constituent proteins.

### *mimi* granules maintain nervous system maturity

**Fig. 5.**
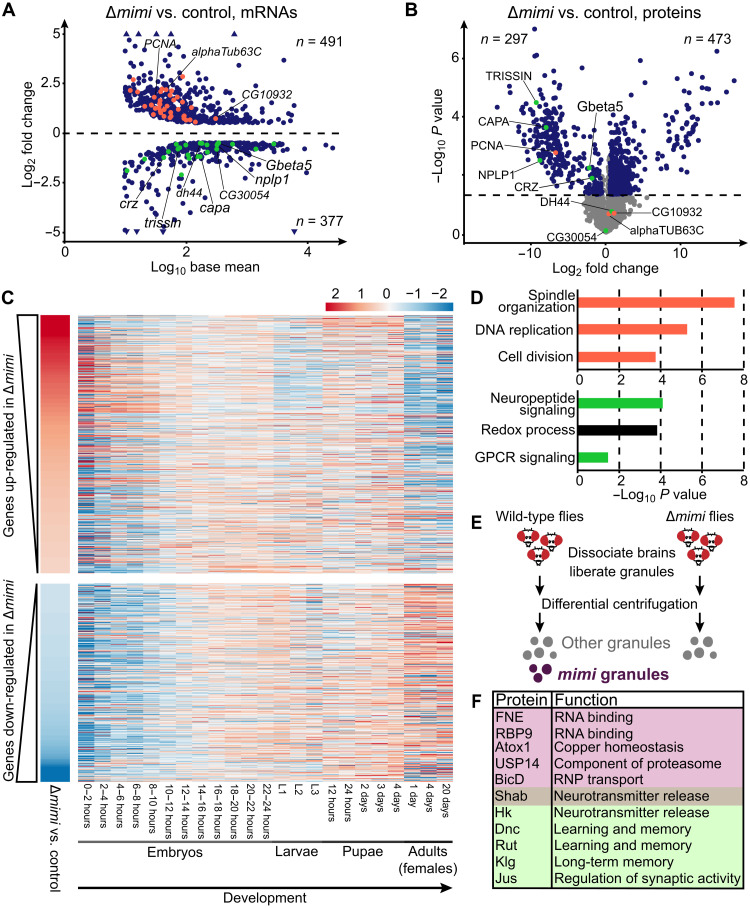
*mimi* granules regulate nervous system maturity and synaptic signaling. (**A** and **B**) Quantification of mRNAs (A) and proteins (B) differentially expressed in Δ*mimi* adult fly heads compared to control (*w^1118^*). Shown are mRNAs with |log_2_ fold change| > 0.5, *P* < 0.05 (two-sided *t* test), and base mean > 10 and proteins with |log_2_ fold change| > 0.5 and *P* < 0.05 (blue). Nonsignificant proteins are in gray. The horizontal line indicates the cutoff *P* < 0.05 (two-sided *t* test). mRNAs represented in (D) are colored accordingly. Genes colored in (A) that were detected in (B) are named. (**C**) Early embryonic genes are preferentially up-regulated in Δ*mimi* heads, whereas adult-specific genes are down-regulated. The heatmap represents the developmental expression of genes up-regulated or down-regulated in Δ*mimi* heads (vertical panel, left). Developmental expression data are from modENCODE (*64*). (**D**) Top 3 enriched biological processes (*P* < 0.05; one-sided EASE score adjusted using the Benjamini-Hochberg method) in up-regulated (top) and down-regulated (bottom) mRNAs in Δ*mimi* heads. (**E**) Workflow to purify neuronal granules including (wild type) or excluding (Δ*mimi*) *mimi* granules. (**F**) Proteins identified as *mimi* granule components based on (E) (selected). Pink shading, components validated by IF; green shading, components involved in synaptic signaling. Shab belongs to both categories.

To study the consequences of *mimi* granule loss on the neuronal transcriptome and proteome, we performed total RNA sequencing and MS analyses on Δ*mimi* heads (Fig. 5, A and B). For roughly half of all significantly deregulated mRNAs, the corresponding protein product was also affected, usually in the same direction (fig. S5A), revealing the broad impact of *mimi* granules on neuronal gene expression.

Most genes down-regulated in Δ*mimi* heads were adult-expressed genes and enriched for synaptic signaling processes characteristic of mature neurons. Moreover, genes normally expressed in early embryonic development, and genes involved in cell proliferation, were enriched among up-regulated mRNAs (Fig. 5, C and D, and table S1). This indicates that *mimi* granules, which form exclusively in adult neurons, as soon as *mimi* is expressed (figs. S2C and S5B), play a role in maintaining the mature state of the nervous system.

### *mimi* granules regulate synaptic signaling

Upon the loss of *mimi* granules, genes involved in neuropeptide-mediated signaling were specifically down-regulated at both mRNA and protein levels. Virtually, all neuropeptides act on G protein–coupled receptors (GPCRs) (*32*). Consistently, the components of GPCR signaling pathways were also affected (Fig. 5, A, B, and D, and table S1), showing that *mimi* granules regulate neuropeptide signaling.

We hypothesized that *mimi* granules contribute to synaptic signaling by harboring signaling molecules. To investigate this possibility, we applied differential centrifugation [adapted from (*33*, *34*)] on lysate from adult fly heads and performed total RNA-seq and shotgun proteomics on the biochemical fractions (Fig. 5E). We obtained fractions enriched in specific RBPs and components of RNP complexes, including *mimi* (fig. S5, C and D, and table S2). To identify the specific components of *mimi* granules, we searched for RNAs and proteins enriched in the granule fraction of control flies compared to Δ*mimi* flies. We identified 352 mRNAs and 297 proteins associated with *mimi* granules (table S3), including, as expected for *mimi* granules, FNE and RBP9. We confirmed the granule localization of several newly identified components by IF: Atox1, Shab, USP14, and BicD localization to *mimi* granules ranged from ~20 to 70% (fig. S5E). Hence, our analysis accurately detects granule components.

The composition of *mimi* granules was rather heterogeneous; Gene Ontology (GO) analysis revealed a modest but significant (*P* < 0.05) enrichment in regulators of neuronal signal transduction and behavior (fig. S5, F and G, and table S3). Notably, we found specific proteins involved in synaptic transmission and memory, including Shaker cognate b and Hyperkinetic (subunits of potassium channels involved in neurotransmitter release) (*35*, *36*), Dunce and Rutabaga (two classic memory-related proteins) (*37*, *38*), and the more recently identified Klingon (Fig. 5F) (*39*). Together, our results show that *mimi* granules are hubs for regulation of RNAs and proteins implicated in neuropeptide signaling, with a potential role in learning and memory.

### Evidence for a function of *mimi* granules in mRNA storage and distribution

To address the molecular effect of *mimi* granules on their resident RNAs and proteins, we asked whether granule components were affected in *mimi* mutants. Granule-associated proteins and RNAs (fig. S6, A to C), but not proteins encoded by granule-associated RNAs (fig. S6, D and E), were significantly more affected by granule loss compared to nongranule components, indicating that *mimi* granules contribute to RNA and protein homeostasis but are unlikely to directly affect translation. However, the global effect of granule loss on the cellular levels of most granule-resident proteins and mRNAs was mild (fig. S6, B, C, and E), suggesting that *mimi* granules do not play a major role in protein/RNA stabilization or degradation. We hypothesize that, instead, *mimi* granules could serve as storage and distribution centers for neuronal RNAs and proteins. Notably, *mimi* granules harbor an assortment of molecules involved in synaptic processes (Fig. 5 and fig. S5, F and G). The regulation of long-distance transport to neuronal projections by pre-assembling and/or stabilizing synaptic RNP transport complexes constitutes a possible molecular function of *mimi* granules.

### Loss of *mimi* granules impairs life span and locomotion in aging flies

**Fig. 6.**
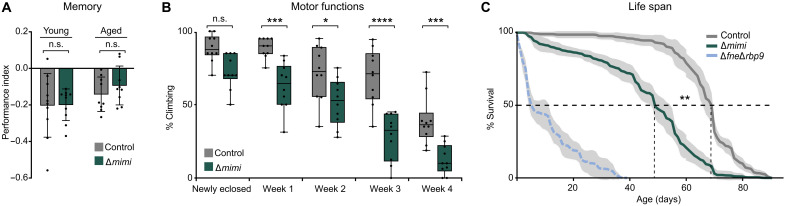
Loss of *mimi* granules impairs life span and locomotion. (**A**) Analysis of aversive memory in Δ*mimi* flies compared to the heterozygous control (*w^1118^*/Δ*mimi*). Learned odor avoidance was tested immediately after aversive conditioning. ^n.s.^*P* = 0.96 (young) and ^n.s.^*P* = 0.33 (aged) (two-sided *t* test). n.s., not significant. (**B**) Age-dependent reduction in climbing performance in Δ*mimi* flies compared to the heterozygous control (*w^1118^*/Δ*mimi*). The percentage of flies climbing above 3 cm within 5 s after startling was scored. One-hundred flies were assessed in 10 biological replicates and two technical replicates for each genotype and time point. Whiskers show the minimum and maximum values. From left to right, ^n.s.^*P* = 0.078, ***P = 5.5 × 10^−4^, *P = 0.014, ****P = 2 × 10^−8^, and ***P = 5.5 × 10^−4^ (one-way analysis of variance adjusted with Holm-Šídák method). (**C**) Life-span shortening in Δ*mimi* flies compared to the heterozygous control (*w^1118^*/Δ*mimi*). Percentage of live flies is represented as a function of time after eclosion. Flies scored: *n* = 80 (Δ*fne*Δ*rbp9*), *n* = 300 (Δ*mimi*), and *n* = 300 (control). Shading represents ±SD of four (Δ*fne*Δ*rbp9*) or six (Δ*mimi* and control) independent replicates. Dotted lines represent half-lives. **P = 0.0022 (two-sided Kolmogorov-Smirnov test).

In both flies and mammals, neuropeptides are crucial regulators of multiple behavioral aspects including learning and memory (*40*, *41*). Because in *mimi* mutants we can uncouple the granule-associated role of RBPs from their other functions in the cytoplasm, we asked whether memory is mediated through *mimi* granules. In an independent, large RNA interference screen study, knockdown of the gene *CG31451* (*mimi*) was found to cause a reduction in memory performance without affecting the fly morphology or activity (*42*). We performed an olfactory learning assay that assesses immediate aversive memory. Δ*mimi* flies did not perform significantly worse than control flies; as the flies aged, memory worsening was notable but not statistically significant (Fig. 6A), potentially due to the low learning score seen in old flies that precludes proper analysis. Our results could not formally prove, but do not exclude, a role of *mimi* granules in learning or memory in aged flies.

Negative geotaxis is an inherent aspect of *Drosophila* locomotor behavior, which can be measured in a climbing assay, an established approach to study motor function in fly models of neurological disease (*43*). In our negative geotaxis assay, Δ*mimi* flies showed reduced climbing performance. Notably, the loss of performance compared to age-matched controls was exacerbated as the flies aged (Fig. 6B). Moreover, we found that the life span of *mimi* mutants was significantly shortened, by ~20%, compared to control flies (Fig. 6C). The decreased longevity and age-dependent degradation of motor functions, in combination, are typical indicators of neurodegeneration (*44*, *45*). Together, our results show that *mimi* granules play an important role in neuronal health and performing vital behaviors.

## DISCUSSION

Cytoplasmic granules are typically thought to be scaffolded by proteins. So far, only one arcRNA, NORAD (noncoding RNA activated by DNA damage), has been described to drive the formation of cytoplasmic condensates: NORAD-Pumilio bodies in nonneuronal cells (*46*). Our study identified the first arcRNA for neuronal granules—*mimi—*a constitutive and essential component of *mimi* biomolecular condensates. The stoichiometry of RBP/RNA interactions is central to phase separation and granule composition, suggesting that many condensates lack a specific scaffolding RNA or protein (*47*). Consistent with this idea, multiple types of neuronal granules undergo dynamic remodeling and contain varying and partially overlapping sets of proteins and RNAs (*48*). Although *mimi* granules share these properties, they distinguish themselves from other neuronal condensates by the presence of a unique identifier, *mimi*. It is therefore conceivable that many neuronal granules, or more generally cytoplasmic condensates, may be architecturally dependent on yet-to-be-discovered arcRNAs. We searched for a functional homolog of *mimi* in mammals using sequence and structure conservation and nELAV (*49*) as well as Staufen (*50*) binding data. So far, we were unable to identify a lncRNA that accumulates in large, Stau1/2-marked granules in mouse or rat neurons. However, the localization of nELAV proteins to the large Stau granules seems to be conserved in mammalian neurons (fig. S4). We propose that strategies to distinguish small from large Staufen granules could have evolved independently: In flies, the sole ortholog Staufen requires not only nELAV proteins but also an arcRNA to form large granules. In mammals, granule identity may be achieved by the preferential accumulation of Stau1 and nELAVs in large granules, while the Stau2 paralog scaffolds small granules.

Our study showed that *mimi* expression and *mimi* granule formation are mutually conditional: In Δ*mimi* flies, the role of the condensate per se can be uncoupled from the individual roles of granule proteins. *mimi* granules mark the transition to the adult nervous system, and their loss caused a shift in gene expression reminiscent of immature cell states. *mimi* granules represent functional nodes at which FNE, RBP9, and Stau interact to maintain the mature neuronal state and perform important neuronal functions such as synaptic signaling. The mechanism of action of *mimi* granules is still not entirely clear. In agreement with our finding that *mimi* granules contain mRNAs and proteins associated with synaptic signaling, *mimi* granules may represent transport intermediates, storing mRNAs destined for travel to neuronal projections via small Stau granules. In addition, compartmentalization of neuronal RBPs may help avoid aberrant function of mislocalized granule-resident proteins or mRNAs, thereby promoting the full deployment of adult neuronal functions.

The functional links between disease-causing mutations and altered granule homeostasis are not well known (*51*). We provide direct evidence that granule hypo-assembly drives neurological dysfunction. *mimi* granule loss results in disrupted expression of neuropeptide-GPCR–mediated signaling components, life-span shortening, and age-dependent decline of motor functions. Neuropeptides are implicated in the pathophysiology of psychiatric disorders such as depression, anxiety, and addiction, as well as of neurodegenerative diseases, particularly those associated with cognitive decline (*52*, *53*). Neuropeptides and GPCRs, through which neuropeptides signal, constitute one of the most widely studied therapeutic targets for the treatment of neurological disorders (*54*). We speculate that compartmentalization of the neuronal cytoplasm through *mimi* granules constitutes a broadly conserved strategy to regulate neuropeptide signaling; studying the underlying mechanisms in mammalian systems will be useful to gain a better understanding of human neurological disease.

## MATERIALS AND METHODS

### Experimental model

Experiments in this study used adult male and female *Drosophila melanogaster*. Flies were raised at 25°C. *w^1118^*, *elav-Gal4*, green fluorescent protein–marked balancer chromosomes, deficiency lines for *rbp9* and *stau*, and the allele *stau^RY9^* were obtained from the Bloomington *Drosophila* stock center (5905, 64349, 458, 4559, 6662, 6663, 8038, 24987, and 10742, respectively). Null alleles for *fne* were obtained from M. Soller (*19*) [*Df(1)fne*^Δ^] and M.-L. Samson (*17*) (*fne^KOZ2^*). The null allele *rbp9^P2690^* (*55*) was obtained from M. Soller. Flies denoted as control are of the genotype *w^1118^* (Figs. 1 to 5 and figs. S1 to S4) or *fne^FLAG^* and *rbp9^FLAG^* (fig. S2K) or *w^1118^*/Δ*mimi* (Fig. 6). Flies denoted as wild type are of the genotype *w^1118^*. Flies denoted as Δ*fne* are of the genotype *Df(1)fne*^Δ^*/fne^KOZ2^* (females) and *Df(1)fne*^Δ^ or *fne^KOZ2^* (males) and Δ*rbp9* flies are of the genotype *rbp9^P2690^/Df(2L)ED206* (males and females). Flies denoted as Δ*fne*Δ*rbp9* are of the genotype *Df(1)fne*^Δ^*/fne^KOZ2^; rbp9^P2690^/Df(2L)ED206* (females) and *Df(1)fne*^Δ^*; rbp9^P2690^/Df(2L)ED206* or *fne^KOZ2^; rbp9^P2690^/Df(2L)ED206* (males). Flies denoted as *stau* deficient are of the genotype *stau^RY9^/Df(2R)BSC483.* Flies denoted as tag-FNE are of the genotype *fne^FLAG^* (*24*) and express an endogenously, N-terminally FLAG-V5–tagged FNE protein. CRISPR-Cas9 genome editing followed the procedure described in (*56*). To generate the *mimi* null mutant Δ*mimi*, two guide RNAs (TGGTAGTACCATGAGGCGTG and AGTGTTAATTGTAAGATCCC) targeted the *CR31451* gene region, generating a 1.2-kb deletion beginning 83–base pair (bp) upstream of the annotated transcription start site (Fig. 2B). To generate tag-RBP9 flies, *rbp9^FLAG^* (expressing an endogenously, N-terminally FLAG-MYC–tagged RBP9 protein), a guide RNA (AGCGTTCGCAAGATGGTCGA) targeted *rbp9* and genome editing used a 3142-bp homology donor (sequence in table S4). To generate the tag-Stau fly strain, *stau^FLAG^* (expressing an endogenously, N-terminally FLAG-V5–tagged Staufen protein), a guide RNA (AGCACAACGTTCATGCCGCC) targeted *stau* and genome editing used a 1440-nucleotide gBlock (Integrated DNA Technologies) as a homology donor (sequence in table S4). To construct the *UAS-mimi* transgene, *mimi-RC* was amplified from fly genomic DNA using the primers (ACCAGGAGCAGTTGAGTATC and CCTGGGATCTTACAATTAACA) and cloned Eco RI/Not I into pUASt-attB (*57*). Constructs were injected, and transgenic flies were generated using targeted integration. All embryo injections were performed by Bestgene Inc. C57BL/6J female mice were bred in-house in the animal facility at the Max Planck Institute of Immunobiology and Epigenetics (MPI-IE) in individually ventilated cages and euthanized by cervical dislocation at 8 weeks of age according to German guidelines. Animal procedures were performed according to the protocols approved by the German authorities and the Regierungspräsidium Freiburg [the sacrificing of animals for scientific purposes according to §4 (3) of the German Animal Protection Act].

### Antibodies and protein detection

For Western blots, rabbit anti-histone H3 (Abcam, Ab1791; RRID:AB_302613), rabbit anti-Staufen (*58*), and peroxidase-conjugated mouse anti-FLAG (Sigma-Aldrich, A8592; RRID:AB_439702) were used at concentrations 1:10,000, 1:2000, and 1:10,000, respectively. Secondary peroxidase-conjugated anti-rabbit antibody (Cell Signaling Technology, #7074; RRID:AB_2099233) was used at 1:2000. For immunohistochemistry, detection was carried out with primary antibodies at concentrations 1:2000 [rabbit anti-Staufen; mouse anti-FMRP; Abcam, ab10299; RRID:AB_297038; rabbit anti-Tral (*59*)], 1:1000 [mouse anti-Cnx99A; Developmental Studies Hybridoma Bank (DSHB), catalog no. Cnx99A 6-2-1; RRID:AB_2722011], 1:100 (mouse anti-MYC; Invitrogen MA1-21316-D550; RRID:AB_2536993; mouse anti-V5; Invitrogen, 37-7500-A488; RRID:AB_2610630; rabbit anti-Staufen1; Abcam, ab73478; RRID:AB_1641030), 1:200 [rabbit anti-Imp (*60*)], 1:500 [guinea pig anti-MAP2 (microtubule-associated protein 2); Synaptic Systems, 188 004; RRID:AB_2138181], and 5 μg/ml (mouse anti-Atox1; DSHB, catalog no. MMC-Atox1-2E6; RRID:AB_2618262; mouse anti-Shab; DSHB, catalog no. K89/34; RRID:AB_2877280; mouse anti-USP14; DSHB, catalog no. AFFN-USP14-9H6; mouse anti-BicD; DSHB, catalog no. anti-Bicaudal-D 1B11; RRID:AB_528102). Mouse anti-HuB (Proteintech, 67097-1-Ig; RRID:AB_2882402), mouse anti-HuC (Santa Cruz Biotechnology, sc-515624), and mouse anti-HuD (Santa Cruz Biotechnology, sc-28299; RRID:AB_627765) antibodies were used at 5 μg/ml. Fluorophore-conjugated secondary antibodies (Invitrogen) were used at concentrations 1:500 (*Drosophila* brains) or 1:750 (mouse primary cultures).

### Immunofluorescence

*Drosophila* adult or larval brains were dissected in phosphate-buffered saline (PBS) and fixed in 4% paraformaldehyde (PFA) in 0.3% PBS–Triton X-100 for 20 min at room temperature. Tissue was permeabilized and rehydrated in 0.3% PBS–Triton X-100 and blocked in blocking solution [5% bovine serum albumin (BSA; Sigma-Aldrich, A9647) and 0.3% PBS–Triton X-100]. Embryos were dechorionated before fixation, then devitellinized, and rehydrated before blocking. Incubation with primary antibodies was carried out in blocking solution for 36 hours at 4**°**C. Brains were rinsed with 0.3% PBS–Triton X-100, washed three times for 20 min with 0.3% PBS–Triton X-100, and incubated with secondary antibodies in blocking solution for 2.5 hours at room temperature. Brains were rinsed and then washed three times for 20 min with 0.3% PBS–Triton X-100, counterstained with 4′,6-diamidino-2-phenylindole (DAPI) and mounted with VECTASHIELD antifade mounting medium (Vector Laboratories, H-1000). Mouse primary cells were fixed in 4% PFA in PBS, permeabilized with 0.2% PBS–Triton X-100, and blocked with 3% BSA for 1.5 hours at room temperature. Incubation with primary antibodies was carried out overnight at 4**°**C followed by secondary antibodies for 1 hour at room temperature. Cells were counterstained with DAPI and mounted with VECTASHIELD antifade mounting medium. Where appropriate, specimens with swapped fluorophores or single-stained controls were prepared to ensure minimal signal bleed-through with the current fluorescence filter settings. All images were acquired with a Zeiss LSM 880 confocal microscope with Fast Airyscan in a sequential scanning mode.

### Image analysis

Image analysis used Fiji software (*61*) with Java 8u181. ZEN (black edition) Imaging Software (Zeiss) was used for image processing. Colocalization between granule components was scored manually by counting the *n*-number of granules or cells showing signal overlap.

### Western blot sample preparation

Adult flies were decapitated, and heads were homogenized in 1× PBS supplemented with protease inhibitor cocktail (Roche, 11873580001). Samples were spun down at 10,000*g* for 3 min to remove the debris. Head lysates were mixed with 4× NuPAGE sample buffer (Invitrogen, NP0007) supplemented with 0.2 M dithiothreitol (DTT) and boiled for 5 min at 95°C.

### RNA purification and RNA-seq

For sequencing of whole head tissue, flies were decapitated and heads were homogenized in QIAzol Lysis Reagent (QIAGEN, 79306). For sequencing of purified neuronal granules, RNA was isolated using TRIzol LS Reagent (Ambion, 10296028). For 3′-seq, RNA samples were prepared as described in the xRIP–quantitative polymerase chain reaction (xRIP-qPCR) input preparation protocol [see “Cross-linking RNA IP followed by reverse transcription q-PCR (xRIP-qPCR)” in Materials and Methods for details]. For all experiments, the total RNA was extracted according to the manufacturer’s instructions and RNA integrity was analyzed using the 2100 Bioanalyzer (Agilent Technologies). Libraries for total RNA-seq were prepared with 725 ng (Fig. 2, A and B) or 100 ng (Fig. 5A) of total RNA using the TruSeq Stranded total RNA (Gold) (Illumina) according to the manufacturer’s instructions. 3′-seq libraries were prepared with 10 ng of total RNA using the QuantSeq 3′-Seq Library Prep Kit REV (Lexogen) according to the manufacturer’s instructions. Paired-end sequencing was performed using the NovaSeq 6000 platform (Illumina) and 51-bp reads (Fig. 2, A and B), 151-bp reads (fig. S2A), or 101-bp reads (Fig. 5A).

### Poly(A) selection

One microgram of purified total *Drosophila* head RNA was used. Poly(A) enrichment was performed with either Oligo d(T)_25_ magnetic beads [New England Biolabs (NEB), S1419S] or Oligo dT beads included in the TruSeq Stranded mRNA Library Prep Kit (Illumina, 20020594) according to the manufacturer’s instructions.

### RNA antisense purification

RNase-free, high-performance liquid chromatography (HPLC)–purified, 5′biotin-conjugated DNA probes with Internal Spacer 18, targeting *mimi-RC*, were obtained from Integrated DNA Technologies (sequences in table S4). Head powder was prepared from *w^1118^* flies. For each sample, 700 mg of head powder was UV-irradiated six times in a Bio-Link BLX 312 crosslinker at 300 mJ/cm^2^. Non–cross-linked powder was prepared as a control. Head powder was homogenized in 6 ml of lysis buffer [50 mM tris (pH 7), 500 mM LiCl, 10 mM EDTA, 5 mM DTT, and 2% lithium dodecyl sulfate (LDS)]. Unless specified otherwise, all buffers were supplemented with protease inhibitor cocktail (Roche, 11873580001) and RiboLock RNase inhibitor (Thermo Fisher Scientific, EO0384). Homogenized samples were incubated at 65**°**C for 2 min at 1100 rpm. Samples were transferred to room temperature for 3 min. After centrifugation (13,750*g*, 4**°**C, 10 min), the top layer and the pellet were discarded. The lower layer containing soluble cellular material was carefully extracted and precleared with high-capacity streptavidin agarose resin (Thermo Fisher Scientific, 20357) at 4°C for 45 min. For hybridization, 1.6 nM biotin-conjugated probes (two sets of probes with two different probes each) were used. Probes were hybridized for 2 min at 65°C and 1100 rpm and then gradually cooled down to 20°C. Samples were diluted 1:1 in lysis buffer without LDS and incubated for 1 hour at 4**°**C with MyOne Streptavidin C1 Dynabeads (Invitrogen, 65002). Beads were washed once in modified lysis buffer (supplemented with 1% LDS and 15 mM DTT) for 5 min at 4**°**C, twice with CHIRP (chromatin isolation via RNA precipitation) buffer (2× SSC, 0.5% SDS, and 15 mM DTT) for 5 min at room temperature, once in urea buffer (1 M urea, 0.5 mM DTT, and no protease or RNase inhibitors) for 5 min at room temperature, and once in ammonium bicarbonate (ABC) buffer (200 mM ABC, 1 mM EDTA, and 15 mM DTT) for 30 min at 37**°**C and 600 rpm and quickly rinsed with 200 mM ABC with 0.01% RapiGest SF surfactant (Waters). Proteins were eluted in 66 μl of elution buffer [50 mM ABC, 0.5 mM MgCl_2_, and 0.01% RapiGest, supplemented with 0.05 U of RNaseI (Thermo Fisher Scientific, EN0601), 0.05 μg of RNaseA (Thermo Fisher Scientific, EN0531), and 13 U of RNaseH (NEB, M0297S)] for 1 hour at 37°C and 900 rpm. The eluates were treated with 0.25 U of Benzonase (Millipore, 70664) for 1 hour at 37**°**C and 900 rpm and adjusted to 10 mM tris-HCl (pH 7.9) and 0.1% SDS before paramagnetic bead–based single pot, solid phase–enhanced sample preparation (SP3) purification as described in (*62*). Peptides were eluted by addition of ultra HPLC grade water (Pierce) in two steps and a final step using 0.1% trifluoroacetic acid pooling the eluates. Pooled eluates were concentrated in vacuo and resuspended in 0.1% formic acid before nano–liquid chromatography–tandem MS (nanoLC-MS).

### Co-IP of FNE and RBP9

Flies (5 g) of the genotype *fne^FLAG^; rbp9^FLAG^* were anaesthetized and blended with five short pulses in a kitchen blender (Philips ProBlend 6) in 400 ml of PBS. The blended mixture was applied onto a stack of sieves with the grid sizes of 710, 425, and 355 μm and subjected to separation with pressurized water. Head tissue was collected on a 100-μm mesh and briefly spun to remove excess liquid. All subsequent steps were performed at 4°C. Heads were homogenized with 12 gentle strokes in a KONTES Tissue Grinder (VWR) (loose pestle) in 3 ml of ice-cold lysis buffer [50 mM Hepes-KOH (pH 7.5), 120 mM NaCl, 2 mM MgCl_2_, and 0.1% NP-40 with protease inhibitor cocktail (Roche, 11873580001)]. After centrifugation through a 40-μm strainer at 400*g* for 2 min, the supernatant was collected and centrifuged at 12,000*g* for 5 min. The middle layer was carefully transferred to a new tube, avoiding the pellet and the top layer. Centrifugation was repeated, and the resulting middle layer (input) was split into 330 μl of samples. Samples were either supplemented with 40 μg of RNaseA (Thermo Fisher Scientific, EN0531) and 25 U of Benzonase (Millipore, 70664) (RNase^+^) or with RiboLock RNase inhibitor (Thermo Fisher Scientific, EO0384) (RNase^−^). Samples were precleared with 50 μl of Pierce control agarose resin (Thermo Fisher Scientific, 26150) for 1 hour. Samples were loaded onto 40 μl of prewashed V5-trap magnetic particles (ChromoTek, catalog no. v5td) or MYC-trap magnetic agarose (ChromoTek, catalog no. ytma) and incubated for 1.5 hours. As a control, one RNase^−^ sample was loaded onto 40 μl of anti-hemagglutinin beads (Thermo Fisher Scientific, 88836). After IP, beads were washed three times for 5 min with 1 ml of ice-cold lysis buffer. After the third wash, samples were transferred into a new tube, the wash buffer was removed, and beads were resuspended in 30 μl of 1× NuPAGE sample buffer (Invitrogen, NP0007) supplemented with 0.2 M DTT and boiled for 5 min at 95**°**C. Eluted proteins were separated on a Western blot together with the input sample.

### Cross-linking RNA IP followed by reverse transcription qPCR (xRIP-qPCR)

One-hundred milligrams of UV–cross-linked head powder (6 × 300 mJ/cm^2^) was homogenized in 1 ml of lysis buffer [200 mM NaCl, 50 mM Hepes-KOH (pH 7.9), 1 mM EDTA, 0.5 mM EGTA, and 0.5 mM DTT] containing 0.2% Triton X-100. Unless specified otherwise, all buffers were supplemented with a protease inhibitor cocktail (Roche, 11873580001) and RiboLock RNase inhibitor (Thermo Fisher Scientific, EO0384). After homogenization, detergents were added to a final concentration of 0.5% SDS and 0.5% Na-deoxycholate, and the samples were incubated 5 min on ice. The homogenate was centrifuged twice at 15,000*g* for 10 min at 4**°**C. Ionic detergents were quenched with 1% NP-40. The processed lysate (750 μl; input) was incubated with 40 μl of anti-FLAG M2 magnetic beads (Invitrogen, M8823) for 1.5 hours at 4°C. Beads were rinsed with lysis buffer containing 0.1% SDS and 0.1% Na-deoxycholate and washed three times for 5 min at 4**°**C with lysis buffer containing 0.1% SDS, 0.1% Na-deoxycholate, and 1% Triton X-100 and twice with lithium chloride buffer [350 mM LiCl, 50 mM Hepes-KOH (pH 7.5), 1 mM EDTA, 1% NP-40, and 0.7% Na-deoxycholate] for 5 min at 4**°**C. Cross-linked protein-RNA complexes were eluted with 120 μl of elution buffer [10 mM tris-HCl (pH 7.4), 150 mM NaCl, 0.1% Triton X-100, and no protease inhibitor] containing FLAG peptide (0.2 mg/ml; Sigma-Aldrich, F3290) for 1 hour at 4**°**C. Eluates and corresponding inputs were subjected to proteinase K (Ambion, AM2546) treatment for 30 min at 50°C and 1100 rpm. RNA was purified with TRIzol LS Reagent (Ambion, 10296028) according to the manufacturer’s instructions. Equal volumes of total RNA from input and IP samples were used for quantitative reverse transcription PCR (RT-qPCR).

### Sequential IF–fluorescence in situ hybridization

Stellaris fluorescence in situ hybridization (FISH) probes with TAMRA dye targeting the *mimi-RC* coding sequence were used (BioCat GmbH). IF-FISH was performed as described in (*63*) with the following modifications. Brains were dissected in PBS; 0.5% PBS–Triton X-100 was used for the preparation of all buffers. Blocking solution contained 2.5% normal goat serum (Abcam, ab138478), 2.5% normal donkey serum (Abcam, ab138579), and BSA (2 mg/ml; Sigma-Aldrich, A9647). Before probe hybridization, brains were incubated overnight with primary antibodies diluted in blocking solution in the presence of Recombinant RNasin Ribonuclease Inhibitor (Promega, N2511). Brains were rinsed three times, washed three times for 15 min with 0.5% PBS–Triton X-100, and incubated for 2.5 hours with fluorescent secondary antibodies diluted in blocking solution. Brains were again rinsed three times and washed three times for 15 min with 0.5% PBS–Triton X-100. Probes were used at a concentration of 5 μM. Hybridization buffer was supplemented with Recombinant RNasin Ribonuclease Inhibitor. Brains were washed two additional times for 10 min with 2× SSC before they were mounted with VECTASHIELD antifade mounting medium (Vector Laboratories, H-1000) and imaged after 24 hours. Where appropriate, specimens with swapped fluorophores or single-stained controls were prepared to ensure minimal signal bleed-through with the current fluorescence filter settings. Images were acquired with a Zeiss LSM 880 confocal microscope with Fast Airyscan in a sequential scanning mode.

### Reverse transcription qPCR

Unless specified otherwise, 500 ng of total RNA was used for RT-qPCR. RT used the iScript gDNA Clear cDNA Synthesis Kit (Bio-Rad). RT-qPCR was performed in a LightCycler 480 II instrument using FastStart SYBR Green Master (Roche). RT-qPCR primer sequences are listed in table S4.

### Purification of native neuronal granules

Nine grams of flies was anesthetized and blended with five short pulses in a kitchen blender (Philips ProBlend 6) in 400 ml of PBS. The blended mixture was applied onto a stack of sieves with the grid sizes of 710, 425, and 355 μm and subjected to separation with pressurized water. Head tissue was collected on a 100-μm mesh and briefly spun to remove excess liquid. All steps of the protocol including centrifugations were performed on ice or at 4**°**C, and all buffers were supplemented with protease inhibitor cocktail (Roche, 11873580001) and RiboLock RNase inhibitor (Thermo Fisher Scientific, EO0384). In a KONTES Tissue Grinder (VWR), 20 strokes with the loose pestle were applied to the sample in 5 ml of lysis buffer [50 mM tris-HCl (pH 7.4), 1 mM EDTA, 150 mM NaCl, and 0.2% Triton X-100], followed by centrifugation through a 100-μm strainer (100*g*, 2 min). The flow-through was processed with 20 strokes using the tight pestle, passed 10 times through a 27-gauge needle, and poured through a 40-μm strainer to remove cuticular debris. The flow-through (tissue lysate) was centrifuged at 1000*g* for 5 min. The resulting supernatant was split in two and centrifuged again at 10,000*g* for 15 min. The pellet (granules 1) was either collected directly or washed by resuspension in 2 ml of 0.3% PBS–Triton X-100/protease inhibitor cocktail/RiboLock followed by centrifugation at 10,000*g* for 10 min (pellet = granules 2). For RNA analysis, both granule fractions were resuspended in lysis buffer without protease inhibitor and subjected to proteinase K (Ambion, AM2546) treatment in the presence of 0.5% SDS for 30 min at 50**°**C and 1100 rpm. Proteinase K treatment was also applied to tissue lysate samples. For proteome analysis, only the fraction granules 2 was resuspended in radioimmunoprecipitation assay buffer [50 mM tris-HCl (pH 8), 150 mM NaCl, 1% NP-40, 0.5% Na-deoxycholate, 0.1% SDS, and 5 mM TCEP], homogenized for 1 min with a mechanical Pellet Pestle Motor (KONTES), incubated for 15 min on ice, and analyzed by nanoLC-MS together with the tissue lysate sample, subsequent to SP3-assisted sample preparation.

### RNA-seq analysis

RNA-seq data from this paper and publicly available data (*64*, *65*) were analyzed using the RNA-seq module from the snakePipes package (*66*). The *Drosophila* annotation from the Ensembl release 96 was used as a reference for mapping and read counting. Differential expression analysis was done using DESeq2 (*67*). Differentially expressed genes were defined on the basis of an absolute log_2_ fold change cutoff of 0.5, base mean > 10, and adjusted *P* < 0.01 (Fig. 2A) or adjusted *P* < 0.05 (Fig. 5A). To identify RNAs specific for *mimi* granules, all genes with base mean > 10 were considered and the log_2_ fold change in granules versus tissue lysate fraction was calculated. To classify an RNA as *mimi* granules component, the following criteria were applied: (i) the difference of the log_2_ fold changes in granules versus tissue lysate fraction for wild type versus Δ*mimi* had to be >0.5 or (ii) the log_2_ fold change in granules versus tissue lysate fraction had to be >0 and significant (*P* < 0.05) only in wild-type condition. For the final classification as *mimi* granule component, an RNA had to fulfill condition (i) or (ii) in the independent analysis of both granule fractions. For 3′-seq analysis, reads were trimmed to remove poly(A) stretches using fastp (*68*) and mapped to the dm6 genome using STAR v2.6.1b (*69*) with default parameters except “--sjdbOverhang 74 --limitBAMsortRAM 60000000000 --alignIntronMax 1.” To remove the signal that likely originates from internal priming, all poly(A) sites that overlap a strand-specific blacklist region containing all genomic positions with more than 70% As in a 10-bp upstream window were removed. Regions with high A density but within 250 bp of annotated transcription end sites were not blacklisted. Remaining single base pair poly(A) sites from all samples with a minimum coverage of 5 reads per sample were clustered together so that sites within 15 bp were merged to one poly(A) cluster.

### Mass spectrometric acquisition

Nanoscale LC-MS analysis was done either on a Q Exactive Plus mass spectrometer coupled to an EASY-nLC 1200 nUHPLC (RAP) or a Q Exactive MS system interlinked to an EASY-nLC1000 nUHPLC (both Thermo Fisher Scientific) as described in (*70*) with modifications detailed below. For the RAP experiment, samples were injected twice (60-min nLC-MS method). The gradient was 5 min, 10%; 40 min, 40%; 4 min, 80%; at the flow rate 250 nl/min. This was followed by a “wash out step” from 80% B buffer at 5 min and a 5-min inverse gradient from 80 to 2% B buffer (flow rate of 450 nl/min). In case of the *mimi* granule study, tissue lysate and granule samples were measured in seven technical replicates using a 120-min nLC-MS method. The gradient was 5% at 0 min, 8% at 5 min, 35% at 90 min, 45% at 10 min, and 80% at 7 min (flow rate of 300 nl/min). This was followed by 5 min inverse gradient from 80 to 2% B buffer (flow rate of 500 nl/min). All measurements were carried out in data-dependent mode using the “sensitive method” (*70*).

### MS data analysis

MaxQuant version 1.6.14.0 using standard parameters was used to identify peptides and final protein identification role-up [both at 1% false discovery rate (FDR)]. MS raw data were searched simultaneously with the target-decoy standard settings against the Uniprot *D. melanogaster* database (Uniprot_reviewed+Trembl including canonical isoforms; downloaded on 5 August 2020) and an in-house curated FASTA file containing commonly observed contaminant proteins. The *mimi* granule study was further analyzed using intensity-based absolute quantification (iBAQ) values. Each protein from a given protein group was assigned a FlyBase ID and included in the analysis as an individual entry. After the analysis, duplicated FlyBase entries were removed. For each of the four conditions (wild-type tissue lysate, wild-type granules, Δ*mimi* tissue lysate, and Δ*mimi* granules), if a single replicate’s iBAQ value was reported missing, then the missing value was imputed on the basis of a nearest neighbor calculation. If two or three of three replicate iBAQ values were missing in a given condition, then these values were instead imputed with a low basement value of 8 to facilitate subsequent processing. Two subsequent filters were applied to remove unreliable proteins from the analysis before assessing for differential expression. A protein was removed if, (i) in wild-type samples, any granules-tissue lysate replicate pair showed missing values in both conditions or (ii) if, in either wild-type or Δ*mimi* samples, a replicate where an iBAQ value was imputed on the basis of the nearest neighbor calculation showed an opposite trend in the pairwise comparison granules versus tissue lysate compared to the replicate with actual reported iBAQ value. To identify candidate *mimi* granule proteins, the relative log_2_ difference in differential expression between granules and tissue lysate samples in the wild-type relative to the Δ*mimi* condition was calculated. iBAQ values were mean normalized using the R package caret (*71*) before differential expression was assessed. In cases where the expression was observed in both wild-type and Δ*mimi* conditions, log_2_ fold change in the granules versus tissue lysate fraction in wild type had to be greater by at least 4.8 than the log_2_ fold change in Δ*mimi*. In cases where no expression was observed in Δ*mimi* samples, only a positive log_2_ fold change expression in granules versus tissue lysate was required in wild type. Proteins that met either of these criteria were considered granule associated. Because some proteins were identified on the basis of <2 unique peptides (as classified by MaxQuant), for proteins shown in Fig. 5F and fig. S5C and proteins indicated as such in tables S2 and S3, we manually aligned (BLASTp search against the *D. melanogaster* whole genome) all identified peptides, which resulted in substantially higher number of peptides that are indeed unique for a given gene. To investigate the effect of *mimi* granule loss on the global proteome, differential protein expression between wild-type and Δ*mimi* tissue lysate samples was assessed. Missing iBAQ values were imputed using the nearest neighbor method and mean-normalized using the R package caret. A two-sample *t* test (two-tailed, equal variance) was run to assess for significant (*P* < 0.05) differential expression between wild-type and Δ*mimi* samples (|log_2_ fold change| > 0.5).

For mimi-RAP experiments, the intersample relative abundance was determined using the MaxQuant MaxLFQ algorithm with enabling the match between runs option (matching time window of 0.5 min). Downstream analysis was carried out using an in-house modified R script using the DEP package as base (*72*). Briefly, contaminants, reverse, and only identified by site entries were filtered out. At least two valid quantitation values in any group (bait or control) were required. Data were vsn (variance-stabilizing normalization)–transformed, and missing values were imputed by drawing values from a normal distribution (width of 0.5, downshift of 1.8). Statistical analysis was done using limma (with trend = TRUE), and the obtained *P* values were corrected for multiple hypotheses by Benjamini-Yekutieli. Differentially enriched proteins were classified by having an adjusted *P* ≤ 0.05 and a |log_2_ fold change| > 0.5 (UV–cross-linked sample versus non–cross-linked control).

### GO enrichment analysis

GO enrichment analysis was performed using DAVID version 6.8 online server. Genes differentially expressed in Δ*mimi* versus control and RNAs classified as *mimi* granule components were queried against the background of all RNAs expressed at base mean > 10. Proteins classified as granule components were queried against the background of proteins detected in either granule or tissue lysate fraction. Significant GO terms were denoted on the basis of *P* < 0.05 calculated using modified Fisher’s exact test (EASE score) with (Fig. 5D) or without (fig. S5, F and G) Benjamini-Hochberg adjustment for FDR correction.

### Behavior assays

#### Survival assay

Flies eclosed within a 2-day window were collected and housed at a density of 25 males and 25 females per bottle. Flies were raised at 25**°**C and transferred into bottles with fresh food every 2 to 3 days. The number of dead and surviving flies was counted at each transfer.

#### Climbing assay

The climbing assay was performed as described in (*73*) with minor modifications. Flies enclosed within a 2-day window were collected and housed at a density of five males and five females per vial. Once a week, starting 3 to 5 days after eclosion, animals were anesthetized on a CO_2_ pad and transferred to an empty vial. After a 20-min recovery period, flies were tapped down to the bottom and the number of flies climbing above the 3 cm mark within the first 5 s after the tap was scored. For each of the 10 biological replicates, two taps separated by 1-min recovery period were performed.

#### Memory assay

All *Drosophila* strains were reared at 25**°**C and 40 to 50% humidity on a standard cornmeal agar food in 12-hour light:12-hour dark cycle. Three- to 5-day-old adult flies were used. Eighty to 100 flies were placed in a 25-ml vial containing standard food and a 20 mm–by–60 mm piece of filter paper for 14 to 22 hours before behavioral experiments. Odors used in all experiments were 4-methylcyclohexanol (MCH) and 3-octanol (OCT) diluted in mineral oil. An odor dilution of approximately 1:103 was used for all experiments (specifically, 7 ml of OCT or 17 ml of MCH in 8 ml of mineral oil). Experiments were performed at 23**°**C and 55 to 65% relative humidity. Aversive olfactory conditioning in the T-maze was conducted as previously described (*74*, *75*). Groups of flies were exposed to the first odor for 1 min [the conditioned stimulus^+^ (CS^+^)] paired with 12 90-V electric shock pulses with 5-s intervals. Following 45 s of clean air, a second odor (the CS^−^) was presented for 1 min without shock. Memory was subsequently assessed by testing flies for their odor preference between the CS^−^ and the CS^+^ in a T-maze (2 min). The performance index was calculated as the number of flies in the CS^+^ arm minus the number in the CS^−^ arm divided by the total number of flies. MCH and OCT were alternately used as CS^+^ or CS^−^, and a single sample, or *n*, represents the average performance score from two reciprocally trained groups. Statistical analyses were performed in GraphPad Prism version 9 (two-sided unpaired *t* test).

### Secondary structure modeling

The taxonomic neighborhood of *D. melanogaster* was screened to find potential homolog sequences. BLASTN (BLAST+ 2.12.0) (*76*) was run against the refseq_genomic database with search results restricted to organism of the Diptera order [National Center for Biotechnology Information (NCBI) taxonomy ID: 7147]. Fourteen candidate sequences for significant hits with full query coverage were extracted. The sequence-structure alignment tool LocARNA version 2.0.0RC8 (*77*) was used in iterative mode to filter candidate sequences for those with compatible sequence and secondary structure. Nine hits with negative alignment scores or mapping to unassembled scaffold regions were discarded. The multiple sequence-structure alignment of the remaining sequences was preprocessed with SelectSequencesFromMSA version 1.0.5 (*78*) predicted by RNAz (2.1) (*79*) to be of structural alignment quality and shows insignificant coding potential with RNAcode version 0.2 (*80*). While some covariant base pairs, representing compensatory mutations to conserve the functional secondary structure, are present, their number is limited due to the phylogenetic scope of the detected potential homologs. RNApuzzler (*81*) and RNAalifold (*82*), both integrated in the ViennaRNA suite version 2.4.13 (*82*), were used to create a visualization of the consensus sequence and secondary structure and of the multiple sequence alignment itself.

### Mouse primary cultures

Eight-week-old female C57BL/6J mice were used. The subventricular zone was dissected, and single-cell suspensions were prepared and cultured as previously described (*83*). Cells were differentiated for 7 days in Dulbecco’s modified Eagle’s medium/F12 medium without glutamine (Gibco, 21331-020) containing 1× B27 supplement (Gibco, 17504-044), 8 mM Hepes (Gibco, 15630-056), 1× GlutaMAX I (Gibco, 35050-038), and antibiotics in 37**°**C and 5% CO_2_.

### Data visualization

The R package ggplot2 or GraphPad Prism version 9 was used for data visualization. Any additional information required to analyze the data reported in this paper is available from V.H. upon request.

### Quantification and statistical analysis

Statistical parameters and tests are reported in the respective figure legends. Statistical tests were performed using DESeq2 (*67*), limma (*84*), caret (*71*), Fisher’s test in R, and GraphPad Prism version 9 for MacOS and *t* test in Microsoft Excel.

### Sample details

In Fig. 1 (A and C), fig. S1 (A, B, and F), Fig. 2 (C and D), figs. S2 (G and K) and S3 (C and E), Fig. 4 (A and D), and figs. S4 (A, B, C, and E) and S5 (B and E), confocal images (IF and IF-FISH) were acquired from three brains (or embryos) per genotype in at least two independent experiments (flies) or from cells isolated from multiple mouse brains. One representative image is shown. Quantification of colocalization between different granule components was scored on the basis of the *n*-number of granules indicated in the respective figure legend. In Fig. 2 (A and B), the total RNA-seq was performed on 20 adult *Drosophila* heads (1-day-old flies, 10 males and 10 females) in five independent replicates (*n* = 5). In Fig. 3B, *mimi* RNA was antisense-purified from thousands of adult *Drosophila* heads in four replicates per condition. In Fig. 3D, tag-FNE, tag-RBP9, and tag-Stau proteins were purified from thousands of adult *Drosophila* heads in three (FNE and RBP9) or four (Stau) replicates per genotype. In Fig. 5 (A, C, and D) and fig. S5 (D and G), for sequencing of neuronal granule components, thousands of adult *Drosophila* heads were used. Tissue lysate samples were prepared and sequenced in three replicates per genotype. Neuronal granules were purified and sequenced in six replicates per genotype (three biological replicates and two granule fractions each). In Fig. 5 (B and F) and fig. S5 (C and F), for LC-MS analysis of neuronal granule components, thousands of adult *Drosophila* heads were used. Both tissue lysates and neuronal granule fractions were analyzed in three independent biological replicates per genotype. In Fig. 6A, memory assays were performed on 200 flies per genotype per replicate. In Fig. 6B, climbing assays were performed on 100 flies per genotype at time point. In Fig. 6C, survival assays were performed on 300 flies per genotype (control and Δ*mimi*) or 80 flies (Δ*fne*Δ*rbp9*).

## Notes

View/request a protocol for this paper from *Bio-protocol*.
