# Supplementary material for: The long noncoding RNA mimi scaffolds neuronal granules to maintain nervous system maturity
Source: Sci Adv. 2022 Sep 28;8(39):eabo5578. doi: 10.1126/sciadv.abo5578 (PMC9519039; doi:10.1126/sciadv.abo5578)
Supplement: Supplementary file 1 — Figs. S1 to S6 [file sciadv.abo5578_sm.pdf]

Science Advances
AAAS

Supplementary Materials for

The long noncoding RNA *mimi* scaffolds neuronal granules to maintain nervous system maturity

Dominika Grzejda *et al.*

Corresponding author: Valérie Hilgers, hilgers@ie-freiburg.mpg.de

Sci. Adv. **8**, eabo5578 (2022)
DOI: 10.1126/sciadv.abo5578

The PDF file includes:

Figs. S1 to S6
Legends for tables S1 to S4

Other Supplementary Material for this manuscript includes the following:

Tables S1 to S4

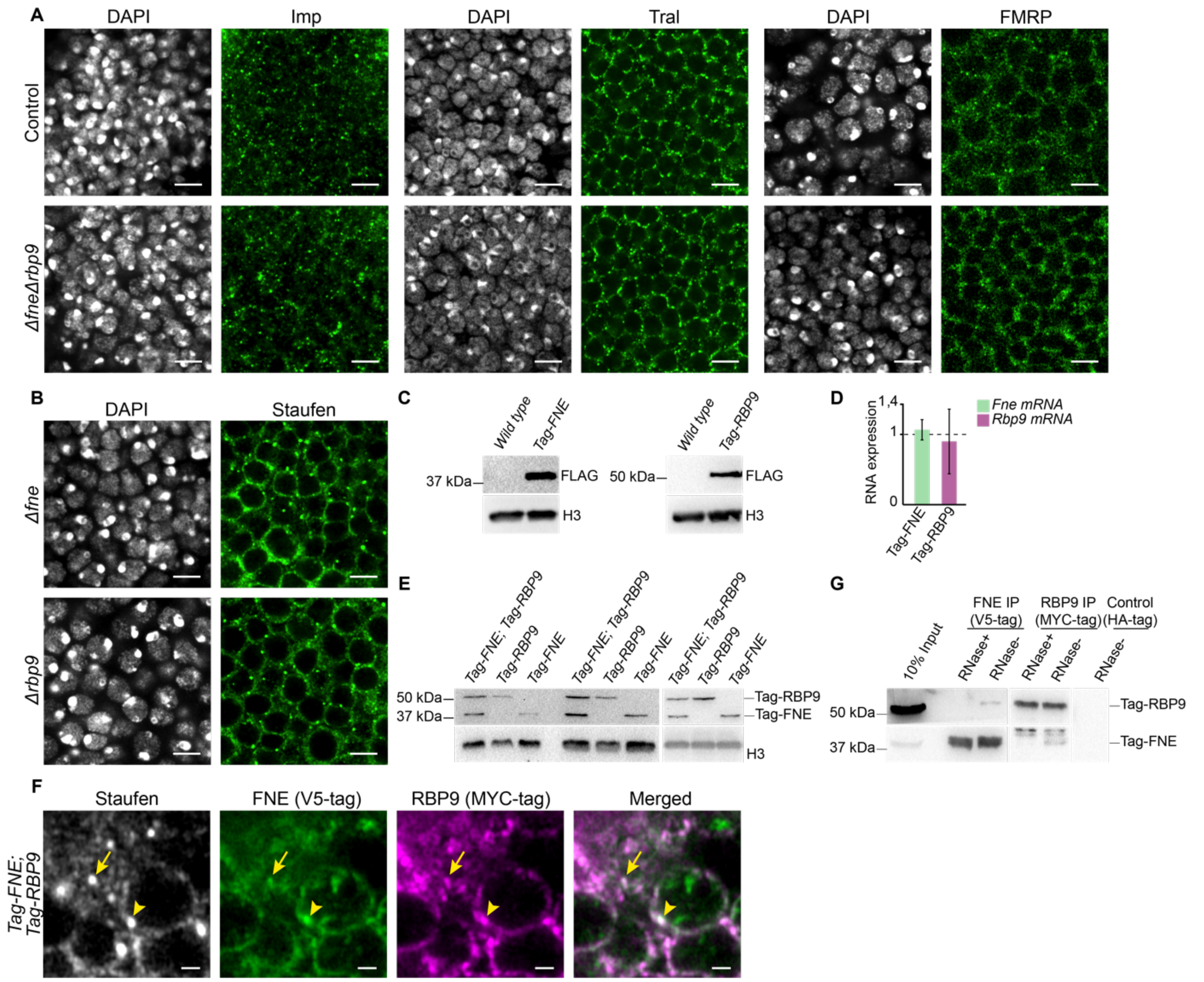

Fig. S1. Several types of neuronal granules are preserved in *ΔfneΔrbp9* fly brains. (**A**) Imp, Tral, and FMRP granules were not detectably affected in *ΔfneΔrbp9* mutants. Shown are single confocal sections of neurons in the midbrain of control (w^{1118}) and *ΔfneΔrbp9* ($Df(1)fne^{\Delta}/fne^{KOZ2}$; $rbp9^{P2690}/Df(2L)ED206$) adult flies. The indicated granule marker proteins were revealed by immunofluorescence. Scale bars: 4 µm. (**B**) Large Stau granules were not affected in *Δfne* or *Δrbp9* single mutants. Shown are single confocal sections of neurons in the midbrain of *Δfne* ($Df(1)fne^{\Delta}/fne^{KOZ2}$) and *Δrbp9* ($rbp9^{P2690}/Df(2L)ED206$) adult flies. Scale bars: 4 µm. (**C**) Western Blot showing expression of tag-FNE and tag-RBP9 in flies expressing endogenously tagged proteins vs. untagged control (w^{1118}), visualized with an antibody against FLAG. Histone H3 serves as a loading control. Protein samples were obtained from six adult fly heads per genotype. (**D**) qRT-PCR quantification of tagged mRNA expression in fly heads expressing endogenously tagged proteins. RNA levels are normalized to *RpL32*; levels in control (w^{1118}) flies are set to the value 1. Error bars represent ±SD of three biological replicates for each genotype. (**E**) Western Blot showing expression of tag-FNE and tag-RBP9 in flies endogenously co-expressing both tagged proteins or only one tagged protein, visualized with an antibody against FLAG. Protein samples were obtained from four adult fly heads per genotype. Three independent biological replicates are shown. (**F**) Single confocal sections of neurons in the midbrain of adult flies expressing endogenously tagged FNE and RBP9 proteins. Stau, tag-FNE, and tag-RBP9 were

visualized with antibodies against Stau, V5, and MYC, respectively. Stau granule signal that overlaps with weak, undefined (arrow) or strong, defined (arrowhead) FNE and RBP9 signal is indicated. Scale bars: 1 µm. (**G**) Immunoprecipitation (IP) in the presence (RNase+) or absence (RNase-) of RNase, using antibodies directed against V5 (FNE), MYC (RBP9), and HA (control) in flies co-expressing the endogenously tagged proteins FLAG-V5-FNE (tag-FNE) and FLAG-MYC-RBP9 (tag-RBP9). The Western Blot was probed with an anti-FLAG antibody.

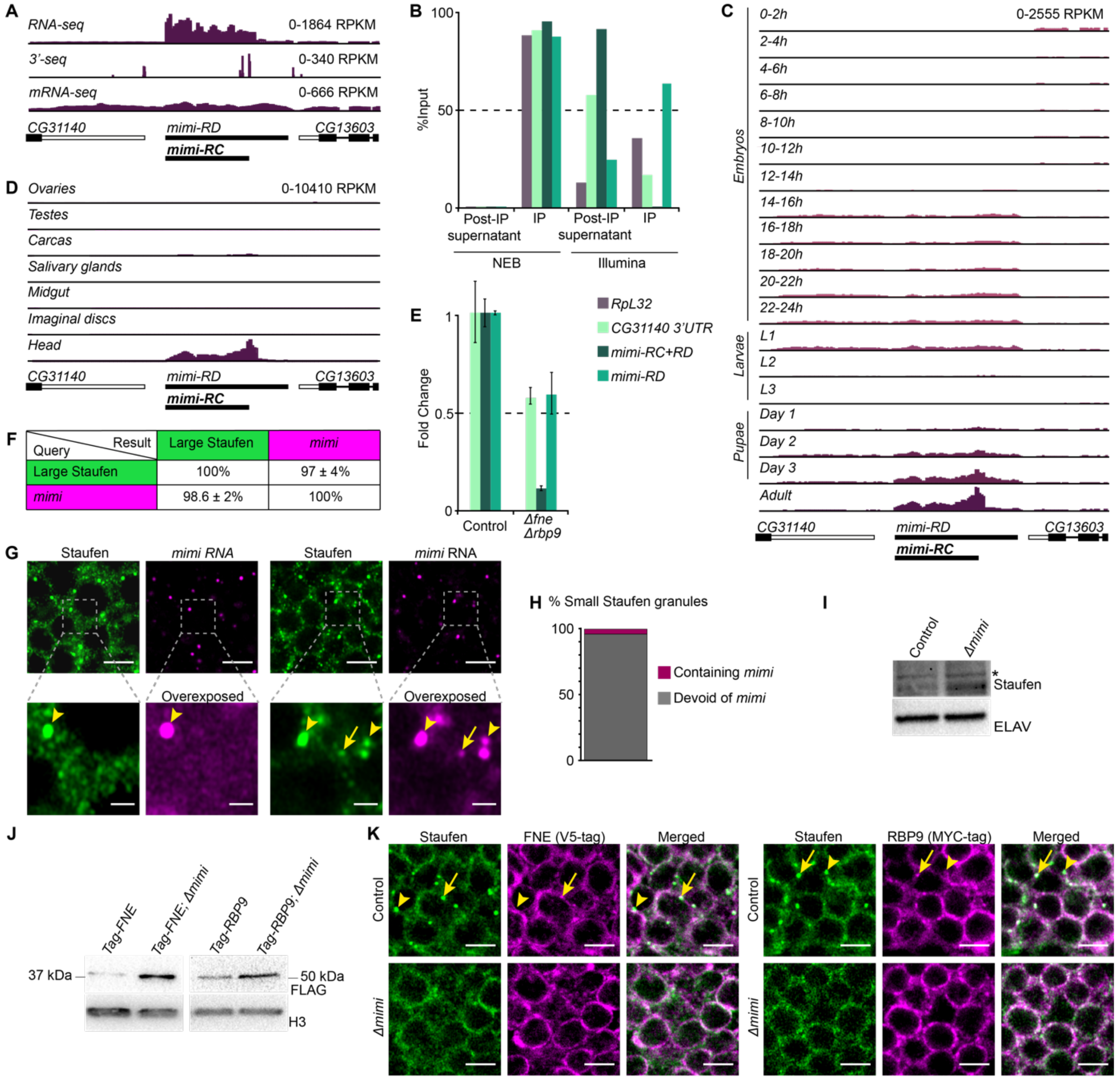

Fig. S2. ***mimi*** **is a polyadenylated lncRNA specifically expressed in the adult fly nervous system.** (**A**) The two isoforms *mimi-RC* and *mimi-RD* were detected by Illumina total RNA-seq and 3'-seq, but not mRNA-seq, which raises the question if *mimi* is polyadenylated. Shown are signal tracks from adult wild-type *Drosophila* heads. RNA-seq indiscriminately sequences all RNAs not captured by ribodepletion. Lexogen 3'-seq selectively amplifies polyadenylated RNAs in a bead-independent manner. mRNA-seq uses oligo-dT beads to purify polyadenylated RNAs for library preparation. (**B**) poly(A) selection on total RNA from fly heads using either the New England Biolabs (NEB) or the Illumina protocol. *mimi-RC* and *mimi-RD* were efficiently captured by NEB, but not Illumina beads. Hence, *mimi* RNAs are polyadenylated, and their elusiveness in Illumina mRNA-seq data is of a technical nature. qRT-PCR quantification of RNA levels of the indicated transcripts in poly(A)-selected samples (IP) and corresponding supernatants. RNA levels are represented as a fraction of input. (**C**) *mimi* RNA expression in fly development. Shown are RNA-seq signal tracks for *mimi* and neighboring genes in the indicated developmental stages. Data

are from modENCODE (*64*) and represent total RNA-seq signal; note that modENCODE mRNA-seq data do not detect *mimi*. (**D**) *mimi* expression is nervous system specific. Shown are RNA-seq signal tracks for *mimi* and neighboring genes in the indicated tissues. Data are from (*65*). (**E**) *mimi-RC* is selectively downregulated in *ΔfneΔrbp9* mutants. qRT-PCR quantification of the indicated transcripts in *ΔfneΔrbp9* (*Df(1)fne*^{Δ}*/fne*^{KOZ2}*; rbp9*^{P2690}*/Df(2L)ED206*) adult heads. RNA levels are normalized to *RpL32*; levels in control (w^{1118}) flies are set to the value 1. Error bars represent ±SD of two biological replicates for each genotype. (**F**) Quantification of the overlap between large Stau granule signal and *mimi* signal; and *vice versa*. (**G, H**) *mimi* RNA is specifically excluded from small Stau granules. (G) Visualization of large and small Stau granules and *mimi*. Stau immunofluorescence was combined with *mimi* RNA FISH. Shown are single confocal sections of neurons in the midbrain of wild-type adult flies. Magnified images show that most large (arrowheads), but not small, Stau granules contain *mimi*. A rare occurrence of a small Stau granule containing *mimi* is shown (arrow). Scale bars: 4 µm (magnified images: 1 µm). *mimi* signal is overexposed in the magnified images for visibility. (H) Quantification of the association of small Stau granules with *mimi*. (**I**) Western Blot comparing Stau protein expression in control (w^{1118}) and *Δmimi* mutant flies. The expected Stau molecular weight is indicated with an asterisk. ELAV serves as a loading control. Protein samples were obtained from six adult fly heads per genotype. (**J**) Western Blot showing tag-FNE and tag-RBP9 in flies expressing endogenously tagged proteins, visualized with an antibody against FLAG. H3 serves as a loading control. Protein samples were obtained from four adult fly heads per genotype. (**K**) Single confocal sections of neurons in the midbrain of adult flies expressing endogenously tagged FNE and RBP9 proteins. Stau, tag-FNE, and tag-RBP9 were visualized with antibodies against Stau, V5, and MYC, respectively. Granules that contain Stau only (arrowhead) or Stau with FNE/RBP9 (arrow) are indicated. Scale bars: 4 µm.

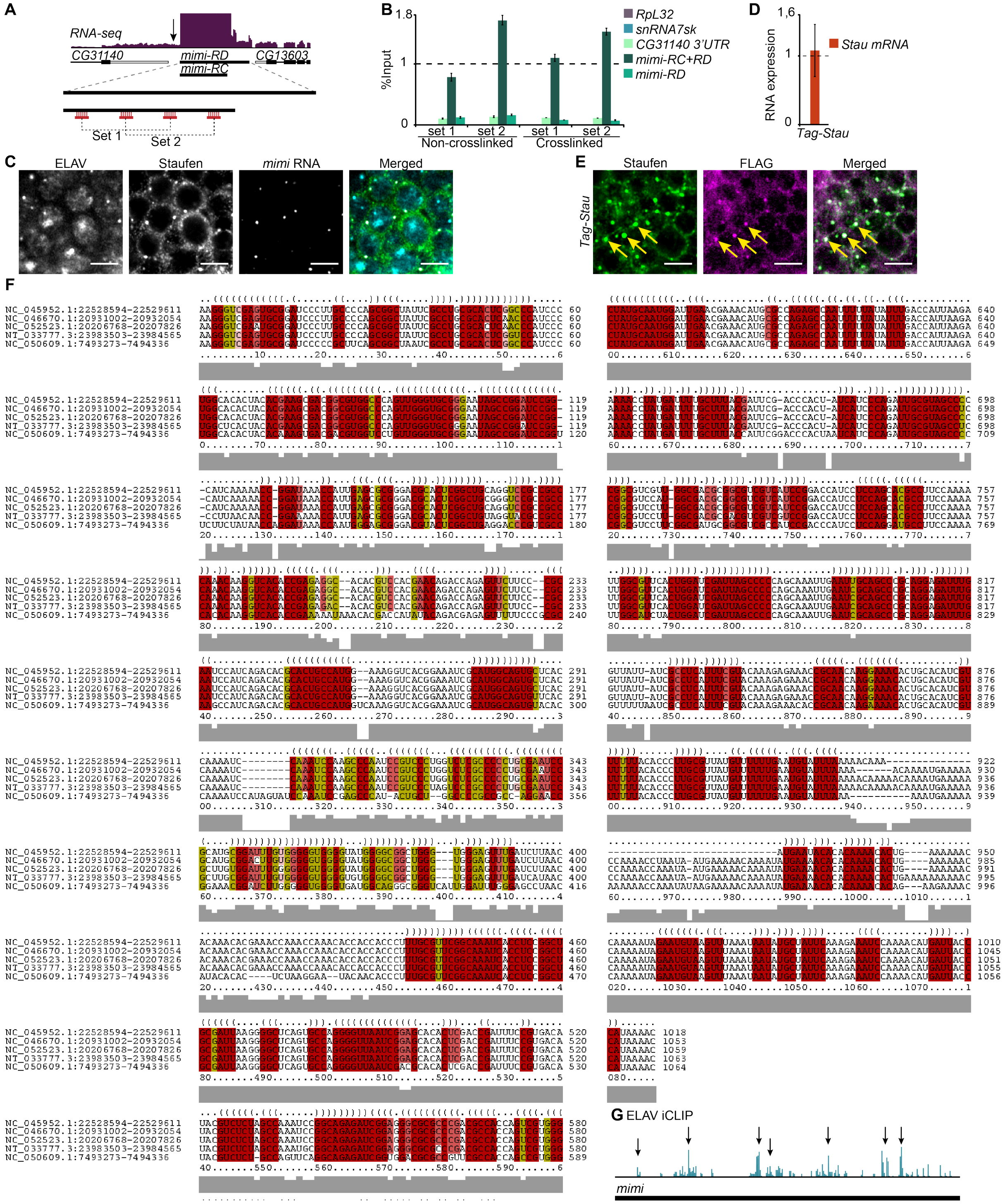

Fig. S3. *mimi* is effectively captured in RNA Antisense Purification (RAP). (**A**) RNA-seq signal tracks of *mimi* and neighboring gene regions. The 3'UTR of *CG31140* extends into the *mimi* locus (arrow), which may cause *CG31140* mRNAs to be targeted by *mimi* probes used for RAP. The region of *mimi* RNA targeted by each antisense DNA probe is illustrated. *mimi-RC* signal (1864 RPKM) extends beyond the shown data range (0-400 RPKM). (**B**) qRT-PCR quantification of RNA levels of the indicated transcripts in purified *mimi* RAP samples. *mimi-RC* was efficiently

and specifically captured in both control (non-crosslinked) and crosslinked conditions, with either set of probes. The percentage of input RNA recovered in the RAP sample is represented for each condition. Error bars represent mean ± SD of two biological replicates. (**C**) ELAV is expressed in neuronal nuclei and was not detected in *mimi* granules. Confocal imaging of ELAV and Stau immunofluorescence combined with *mimi* RNA *in situ* hybridization in neurons in the midbrain of wild-type adult flies. Scale bars: 4 µm. (**D**) qRT-PCR quantification of *stau* mRNA levels in heads of tag-Stau flies. RNA levels are normalized to *RpL32*; levels in control (w^{1118}) flies are set to the value 1. Error bars represent ±SD of three biological replicates. (**E**) Endogenously tagged Stau is expressed in the expected pattern in Drosophila brains. Shown are single confocal sections of neurons in the midbrain of adult flies expressing FLAG-V5-tagged Stau. The protein was visualized with antibodies against Stau and FLAG. Arrows indicate exemplary granules where Stau signal overlaps with FLAG signal. Scale bars: 4 µm. (**F**) Multiple *mimi* sequence structure alignment of homology search hits in the taxonomic neighborhood of *Drosophila melanogaster*. Shown are sequences of potential Diptera homologs. Species: *Drosophila sechellia, Drosophila mauritiana, Drosophila simulans, Drosophila melanogaster, Drosophila subpulchrella* (from top to bottom). Covariance is color-coded from dark red (low covariance) to green (high covariance). (**G**) ELAV iCLIP tracks for *mimi*. The main crosslinking site from each iCLIP cluster is marked with an arrow and represented as ELAV protein binding to *mimi* RNA in Figure 3E. Data are from (*24*).

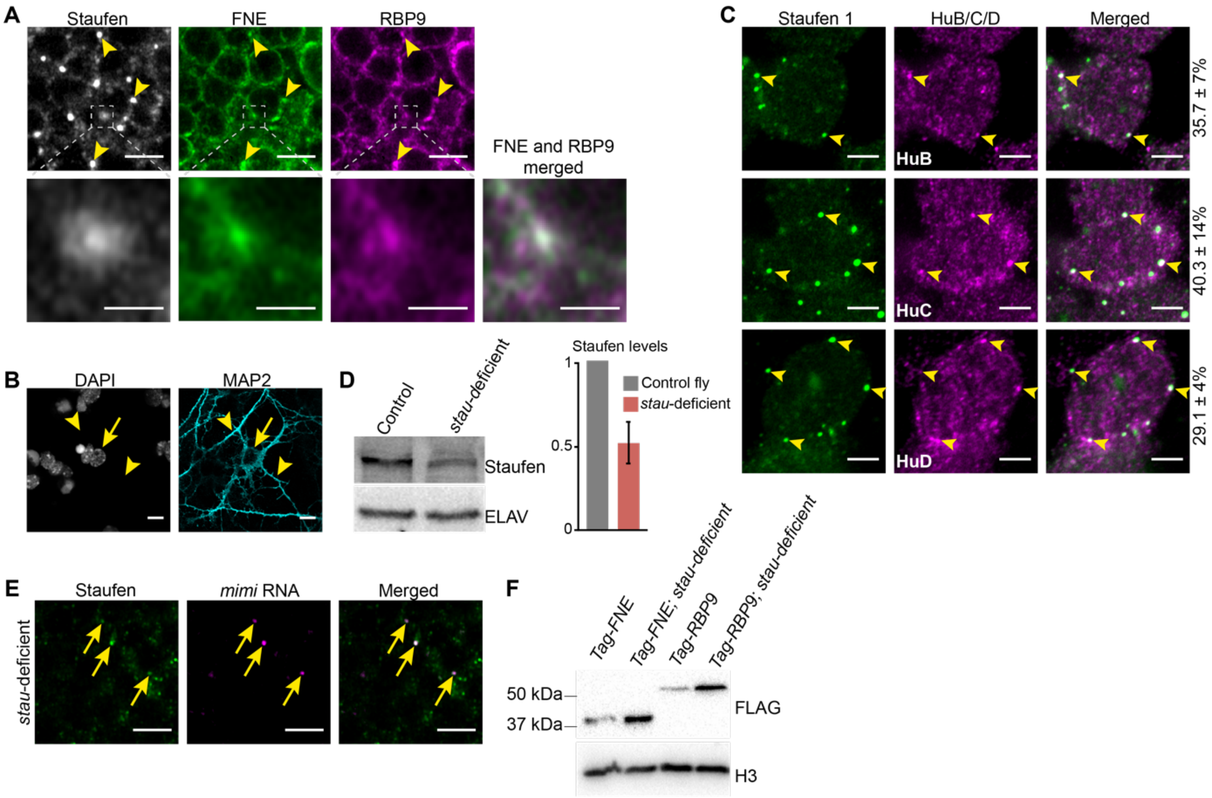

Fig. S4. FNE and RBP9 participate in *mimi* granule assembly. (**A**) Visualization of the association of diffuse (magnification) and condensed (arrowheads) Stau foci with FNE and RBP9. Shown are single confocal sections of neurons in the midbrain of adult flies expressing endogenously tagged FNE and RBP9 proteins. Stau, tag-FNE, and tag-RBP9 were visualized with antibodies against Stau, V5, and MYC, respectively. Quantifications are shown in Figure 4B. Scale bars: 4 µm (magnified images: 1 µm). (**B**) Subventricular zone (SVZ) primary cultures differentiate into neuronal lineage and form *de novo* connections. Shown are cultured cells from the SVZ of mouse brain that were differentiated for 7 days and stained with a MAP2 antibody (neuronal marker). Nuclei were counterstained with DAPI. Differentiated SVZ cultures acquire neuronal morphology with branched projections (arrowhead) protruding from the soma (arrow). Scale bars: 8 µm. (**C**) Mouse Stau1 granules are associated with nELAV/Hu proteins. Shown are single confocal sections of differentiated neurons cultured from the subventricular region of mouse brain. Stau1 granules containing the indicated nELAV/Hu protein are denoted with arrowheads. Quantifications are shown on the right. Granules scored: n=201, n=197 and n=298 for HuB, HuC and HuD, respectively. Scale bars: 4 µm. (**D**) Representative Western Blot showing Staufen expression in control (w^{1118}) and *stau*-deficient flies. Protein signal from *stau*-deficient heads was quantified relative to control flies (bar graph). Error bars represent ±SD of four biological replicates. ELAV serves as a loading control. Protein samples were obtained from three adult fly heads per genotype. (**E**) Confocal imaging of neurons in the midbrain of *stau*-deficient adult flies. Residual Stau granules overlapping with *mimi* signal are indicated (arrows). Scale bars: 4 µm. Staufen was detected with a secondary antibody coupled to Alexa Fluor 488 (Alexa Fluor 555 shown in Fig. 4D). (**F**) Western Blot showing expression of tag-FNE and tag-RBP9 in flies of the indicated genotypes, visualized with an antibody against FLAG. Histone H3 serves as a loading control. Protein samples were obtained from six adult fly heads per genotype.

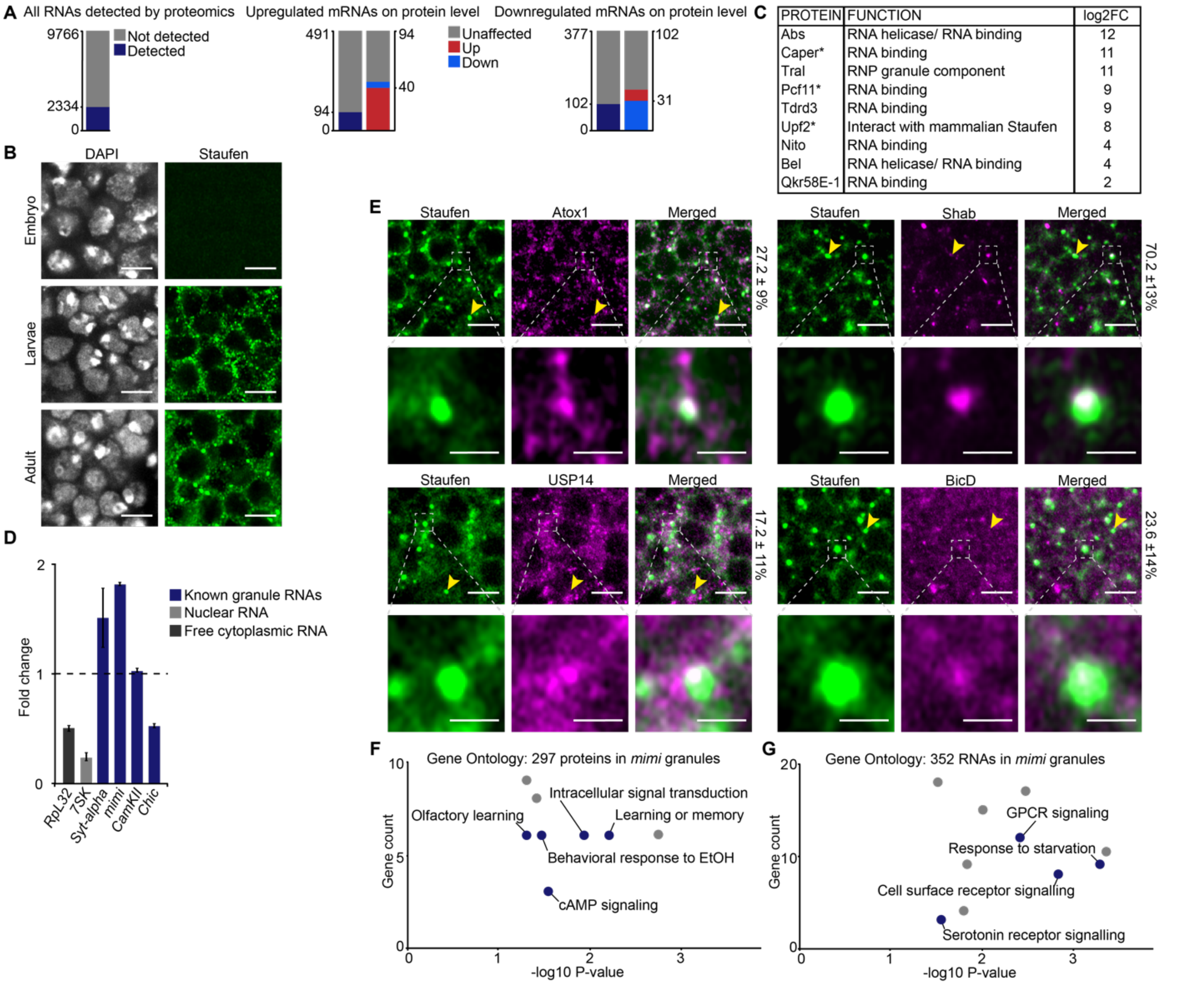

Fig. S5. *mimi* granules influence nervous system maturity and regulate synaptic signaling. (**A**) Deregulation of the *Δmimi* transcriptome is largely reflected in the proteome. The fraction of the total, up- or downregulated RNAs encoding proteins that were detected in the shotgun proteomics experiment are shown in dark blue (24%, 19% and 27% respectively). Red and light blue represent the fraction of up- or downregulated proteins, respectively, among the proteins encoded by up- (middle panel) or downregulated (right panel) mRNAs. (**B**) *mimi* granules are exclusively found in the adult nervous system. Shown is Stau immunofluorescence in single confocal sections of neurons in the embryonic ventral nerve cord, larval brain and adult midbrain. Nuclei are counterstained with DAPI. Scale bars: 4 µm. (**C**) RBPs and known granule components were enriched among proteins recovered in the granule fraction. Shown are selected proteins significantly enriched in wild-type granules (log2 fold change >0.5; *P*<0.05; two-sided paired t-test). The log2 fold change represents enrichment in the granule fraction vs. tissue lysate. Proteins that were not consistently identified based on >2 unique peptides in all 3 replicates are marked with an asterisk (see methods). (**D**) qRT-PCR quantification of RNA levels of the indicated genes in granules purified from head tissue of control (w^{1118}) flies. The RNA enrichment in the granule fraction is represented as fold-change over tissue lysate for each gene. *Syt-alpha* and *mimi* were enriched in granules, whereas other known neuronal granule mRNAs, such as *CamKII* and *chic* were not enriched, or even depleted. *RpL32*, a cytoplasmic mRNA, and *7SK*, a nuclear RNA, were

depleted in purified granules. Error bars represent ± SD of three biological replicates. (**E**) Validation of Atox1, Shab, USP14, and BicD as proteins associated with *mimi* granules. Shown are single confocal sections of neurons in the midbrain of adult flies. The indicated granule proteins were revealed by immunofluorescence. Magnified cutouts and arrowheads indicate, respectively, granules containing and devoid of each granule protein. Quantifications of the percentage of *mimi* granules containing each protein are shown. Granules scored: Atox1, n= 297; Shab, n= 1496; USP14, n= 284; BicD, n= 285. Scale bars: 4 µm (magnified images: 1 µm). (**F, G**) Gene Ontology (GO) classification of biological processes for (F) proteins (n=297) and (G) RNAs (n=352) enriched in *mimi* granules. The number of genes associated with each term is plotted as a function of its *P* value (P<0.05; one-sided EASE score). Selected terms related to neuronal function are labeled in dark blue. The full lists of significantly enriched biological processes are shown in Table S3.

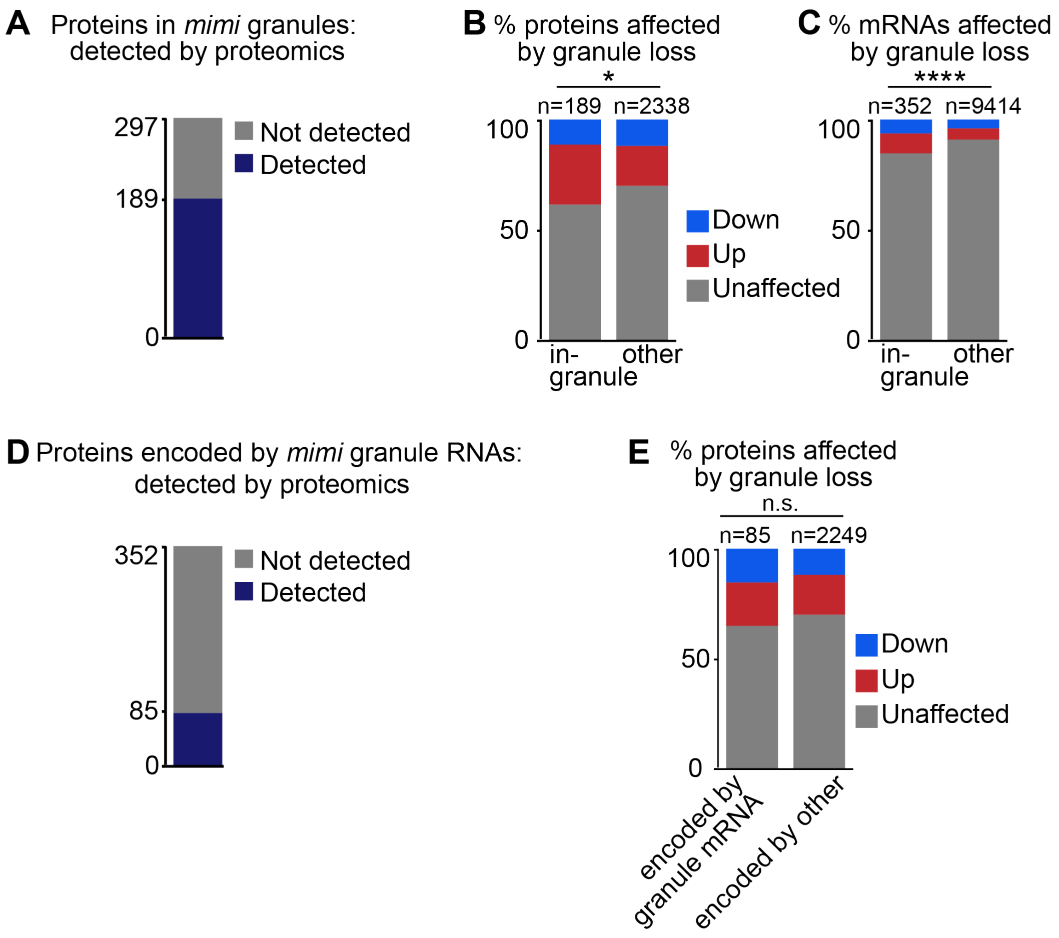

Fig. S6. Molecular function of *mimi* granules. (**A**) Number of proteins residing in *mimi* granules that were detected by proteomics in head tissue lysate. (**B, C**) Loss of *mimi* granules affects expression of granule components. Shown is the fraction of affected granule-resident (in-granule) proteins (B) and mRNAs (C), in comparison to non-granule-resident (other). *P=0.026, ****P=7.7e-5 (two-sided Fisher's exact test). (**D**) Number of proteins encoded by mRNAs residing in *mimi* granules that were detected by proteomics in head tissue lysate. (**E**) Loss of *mimi* granules does not significantly affect protein output from granule-resident mRNAs. Shown is the fraction of affected proteins encoded by granule-resident mRNAs compared to other mRNAs. $^{n.s.}P$=0.34 (two-sided Fisher's exact test).

Supplementary Datasets:

Table S1. GO term analysis of mRNAs significantly affected in *Δmimi* mutants.

Table S2. mRNAs and proteins found enriched in the granule fraction.

Table S3. mRNAs and proteins identified as components of *mimi* granules with GO term analysis.

Table S4. Oligonucleotides used in this study.
